# Exosome-sheathed porous silica nanoparticle-mediated co-delivery of 3,3′-diindolylmethane and doxorubicin attenuates cancer stem cell-driven EMT in triple negative breast cancer

**DOI:** 10.1186/s12951-024-02518-0

**Published:** 2024-05-25

**Authors:** Rupali Sarkar, Souradeep Biswas, Rituparna Ghosh, Priya Samanta, Shampa Pakhira, Mrinmoyee Mondal, Yashaswi Dutta Gupta, Suman Bhandary, Prosenjit Saha, Arijit Bhowmik, Subhadip Hajra

**Affiliations:** 1 Department of Cancer Chemoprevention, Chittaranjan National Cancer Institute (CNCI), 37, S.P. Mukherjee Road, Kolkata, West Bengal 700 026 India; 2 Department of Biological Sciences, School of Life Science and Biotechnology, Adamas University, Kolkata, 700126 West Bengal India

**Keywords:** TNBC, Cancer stem cell, EMT, 3,3′-diindolylmethane, Doxorubicin, Exosome

## Abstract

**Background:**

Therapeutic management of locally advanced and metastatic triple negative breast cancer (TNBC) is often limited due to resistance to conventional chemotherapy. Metastasis is responsible for more than 90% of breast cancer-associated mortality; therefore, the clinical need to prevent or target metastasis is immense. The epithelial to mesenchymal transition (EMT) of cancer stem cells (CSCs) is a crucial determinant in metastasis. Doxorubicin (DOX) is the frequently used chemotherapeutic drug against TNBC that may increase the risk of metastasis in patients. After cancer treatment, CSCs with the EMT characteristic persist, which contributes to advanced malignancy and cancer recurrence. The latest developments in nanotechnology for medicinal applications have raised the possibility of using nanomedicines to target these CSCs. Hence, we present a novel approach of combinatorial treatment of DOX with dietary indole 3,3′-diindolylmethane (DIM) which is an intriguing field of research that may target CSC mediated EMT induction in TNBC. For efficient delivery of both the compounds to the tumor niche, advance method of drug delivery based on exosomes sheathed with mesoporous silica nanoparticles may provide an attractive strategy.

**Results:**

DOX, according to our findings, was able to induce EMT in CSCs, making the breast cancer cells more aggressive and metastatic. In CSCs produced from spheres of MDAMB-231 and 4T1, overexpression of N-cadherin, Snail, Slug, and Vimentin as well as downregulation of E-cadherin by DOX treatment not only demonstrated EMT induction but also underscored the pressing need for a novel chemotherapeutic combination to counteract this detrimental effect of DOX. To reach this goal, DIM was combined with DOX and delivered to the CSCs concomitantly by loading them in mesoporous silica nanoparticles encapsulated in exosomes (e-DDMSNP). These exosomes improved the specificity, stability and better homing ability of DIM and DOX in the in vitro and in vivo CSC niche. Furthermore, after treating the CSC-enriched TNBC cell population with e-DDMSNP, a notable decrease in DOX mediated EMT induction was observed.

**Conclusion:**

Our research seeks to propose a new notion for treating TNBC by introducing this unique exosomal nano-preparation against CSC induced EMT.

**Graphical Abstract:**

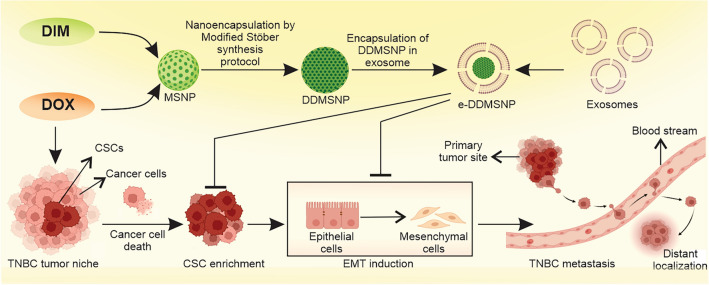

**Supplementary Information:**

The online version contains supplementary material available at 10.1186/s12951-024-02518-0.

## Introduction

TNBC is a heterogenous subtype of breast cancer that mostly happens in premenopausal young women less than 40 years of age [1]. It has a poor prognosis and comprises approximately 15 to 20% of all breast carcinomas. TNBC patients have lower survival time compared with other subtypes of breast cancer. Although, in TNBC the mortality rate is 40%, approximately 46% of patients experience a relapse or distant metastasis in the first 3–5 years after diagnosis. There are several breast cancer treatments available, surgically removing the original tumor is frequently necessary to manage the advanced illness. Unfortunately, research from both experimental and clinical settings has indicated that cancer surgery may raise the chance of metastasis and recurrence. Cancer cells propagate from the primary tumor to distant organs through a complicated process called metastasis, which is divided into several phases such as the tumor cells’ separation from the primary tumor, migration, invasion, intravasation, survival in the vasculature, extravasation, and secondary site colonization [2]. A disruptive generation of genetic and epigenetic change of tumor cells (intrinsic factors) and a chaotic milieu (extrinsic signals) turn cancer cells into metastatic ones during this metastatic cascade. The primary issue in breast cancer research is determining the extrinsic and intrinsic signals that cause breast cancer metastasis [3].

Cancer stem cells (CSCs) are a subset of the tumor mass which have the capability of self-renewal, differentiation into other cell types and are responsible for cancer initiation, progression and metastasis [4]. It is hypothesised that CSCs remain dormant throughout the chemotherapy, allowing them to continue to generate cancer after the treatment has ended [5]. Dormancy is a condition in which tumor cells are hidden and unnoticeable throughout the development of the tumor or following therapy and this condition can be observed in TNBC. The epithelial to mesenchymal transition (EMT) is an important phase in metastasis because it entails a change in gene expression patterns that leads in cells with greater mobility. Following their detachment from the primary sites, CSCs that have undergone EMT induction must go through the lymphatic and circulatory systems to disseminate [6, 7]. Therefore, there is growing proof of a correlation between these EMT and CSCs [8]. Along with these, the biology of cancer metastasis may be better understood by in-depth molecular and functional studies of EMT induction in CSCs. Thus, discovering alternative therapy modalities for targeting CSCs offer a promising strategy for fighting and eliminating cancer metastasis. The primary form of treatment for TNBC patients is conventional cytotoxic systemic chemotherapy. Primarily, they are susceptible to conventional chemotherapy but the initial susceptibility to this treatment does not correlate with overall survival in TNBC patients who have acquired complete remission.

Among systemic chemotherapeutic drugs, doxorubicin (DOX), an anthracycline antibiotic, is commonly used as a first line treatment in TNBC patients. It causes topoisomerase II inhibition, free radical production, and intercalation of DNA and proteins. However, the clinical use of DOX is limited due to the development of myocardial toxicity in patients as well as cytotoxic effects on normal cells, leading to unwanted side effects that may substantially impact patient health and quality of life. DOX is so important in cancer chemotherapy; it is crucial to lessen its toxicity to normal cells. Concurrent administration of antioxidants or other free radical scavengers can help to achieve this goal. According to epidemiological research, consuming cruciferous vegetables may help to prevent various chronic diseases, particularly cancer. One of the key constituents of cruciferous vegetables like cabbage, cauliflower, radish, mustard greens, turnip, broccoli is 3,3′-diindolylmethane (DIM) which has the ability to curb the effect of DOX [9]. Chu et al. reported that DIM is able to target CSCs in various human cancers [10]. According to Thekkekkara et al. DIM is a nontoxic compound and has radioprotective potential due to its propensity to scavenge free radicals and disrupt many signalling cascades that result in cell damage from ionising radiation [11]. It is also fascinating that certain compounds that have been demonstrated to have a calming impact on the DOX-induced hepatotoxicity, such as extracts from medicinal plants, natural goods, and chemicals [12]. In this respect DIM could be an effective combination with DOX that may increase anti-TNBC efficacy of it. Along with that, it is intriguing to find out whether this combinatorial therapy could be an effective measure for treating CSC mediated EMT in TNBC. Poor drug solubility, half-life, and drug resistance as a result of insufficient drug binding with the particular ligands of the tumors are the main causes of limitations in anticancer medication potency [13]. Testing of nano-formulations such as hydrogels, microneedle patches, biodegradable polymers, microspheres, and nanoparticles have been conducted in an effort to discover a suitable solution to all of these clinical issues [14]. Although the effectiveness of the nano-formulations was demonstrated in pre-clinical studies, their use in clinical trials has been hampered by toxicity and off-target effects. All of the aforementioned challenges validate the development of new and enhanced nanocarriers with suitable traits including low toxicity, extended half-life, and biocompatibility. To target specific ligands creating engineered nanocarriers can be a solution. To fulfil these needs, researchers invented extracellular vesicles, exosomes, as the delivery system for various chemotherapeutics. Exosomes are membrane-derived lipid vesicles with a diameter of ~ 40–200 nm that are secreted by several cell types and exist both in vitro and in vivo [15]. Along with the properties like contribution to homeostasis and development of disease including cancer and neurodegenerative disorders, these vesicles are being explored for better delivery of therapeutic payloads to specific cells or tissues, harnessing their endogenous tissue homing capability [15]. Exosomes are effective delivery agents with a small size and increased biocompatibility compare to the other nanoformulations.

In light of the above considerations, we synthesized DIM and DOX loaded mesoporous silica nanoparticles encapsulated in exosome (e-DDMSNP) for targeting cancer cells and CSCs. It should be noted that upon DOX administration the expression of different EMT markers like N-cadherin, Snail, Slug and Vimentin have been up-regulated whereas expression of E-cadherin level is decreased in CSC populations. To confirm this in vitro study of EMT induction, metastasis in tumor-bearing mice upon DOX treatment is monitored. Since the breast cancer cell marker BRCA1 is overexpressed in the lung of mice receiving DOX treatment, it has been observed that DOX increases the risk of metastasis. On the other hand, after treating with the e-DDMSNP, CSC mediated sphere formation rate were notably impaired, indicating the inhibition of stemness. More significantly, in the in vivo model that was treated with the e-DDMSNP, the mice exhibited the suppressed tumor initiation, progression, the median survival time and metastasis. Therefore, this e-DDMSNP mediated CSCs-targeting strategy may pave a new avenue for preventing tumor metastasis.

## Materials and methods

### Synthesis of DIM and DOX loaded PEG-PLGA based mesoporous silica nanoparticle

The mesoporous silica nanoparticles (MSNPs) were synthesized by modifying the procedure reported by Stӧber et al. [16]. In a solution of cetyl trimethyl ammonium bromide (CTAB) and 10% ammonium acetate, 1 N sodium hydroxide was added dropwise to adjust the pH to approximately 11. This solution was then magnetically stirred for 1 h at 75 °C. After that, tetraethyl orthosilicate (TEOS) was added to the solution immediately followed by drop wise addition of 3-aminopropyl triethoxysilane (APTES) and stirred for another 2 h at room temperature. Then the solution was subjected to centrifugation and the resultant pellet was redispersed in methanol. To remove excess surfactant molecules methanol wash was conducted by repeated centrifugations. The supernatant was then discarded, and MSNPs were allowed to air dry.

After that, 20 mg of MSNPs were dispersed in 4 ml of double distilled water (dH_2_O) in a round-bottom flask using a magnetic stirrer. 1 mM DIM solution was added drop wise and the solution was stirred for 2–3 h. The solution was then collected in microcentrifuge tubes and centrifuged and the pellet of DIM encapsulated in MSNP (NDIM) was collected and allowed to air dry. In case of DOX encapsulated in MSNP (NDOX), using a magnetic stirrer, 20 mg of MSNPs were dissolved in 4 ml of dH_2_O in a round bottom flask. 1 mM of DOX solution was added drop by drop in the solution and agitated for 2–3 h. Then it was again centrifuged and the supernatant was discarded. The pellet of NDOX was allowed to air dry.

Other than these, 25 mg of MSNPs were dispersed separately in 10 ml of ethanol and DOX solution (1 mM) was added dropwise and stirred for 2 h in dark. The surface of the DOX-loaded MSNPs was functionalized with amino groups by adding 20 µl of 3-aminopropyl triethoxysilane (APTES) solution that was previously prepared in ethanol. The resulting mixture was allowed to stir for another 6 h. The DOX-loaded amino functionalized MSNPs (DOX-fMSNPs) were isolated by centrifugation. The pellet was allowed to air dry. The DOX-fMSNPs were re-dispersed in 4 ml of dH_2_O in a round-bottom flask. Then 1 mM DIM solution was added dropwise to this solution and simultaneously, 100 µl of 1 mg/ml PEG-PLGA solution was added to the mixture and continued to be stirred for another 2 h. Following this, the solution was centrifuged and the pellet was allowed to air dry naturally to generate the final product PEG-PLGA encapsulated DIM and DOX loaded MSNPs (DDMSNPs).

### Characterization of empty MSNPs and DDMSNPs

For this purpose, the samples were prepared by pressing it into KBr pellets in a ratio of 2:8. It was then scanned between 4000 and 400 cm^−1^ with a resolution of 4 cm^−1^. Morphological characteristics of the MSNPs and DDMSNPs were observed by scanning electron microscope (SEM) model FEI Quanta 250 (USA). Before recording the images, the samples were sputter coated with gold and then observed by SEM. The concentration of DIM and DOX was determined using UV–Vis spectrophotometer (Shimadzu UV 1800, Japan). To determine the morphology of MSNPs and DDMSNPs, JEOL JEM-2100 transmission electron microscope (TEM) (JEOL, Inc., Peabody, MA, USA) was used at an acceleration voltage of 300 kV. A drop of nanoparticle suspension was dispersed in DI water, lyophilized and mounted on a thin film of amorphous carbon deposited on a copper grid (300 meshes). After that, the grids were dried in clean condition and examined directly with the TEM. Nano Zetasizer (Malvern Instruments, Malvern, UK) was applied to perform dynamic light scattering (DLS) to identify the size of MSNPs. The most important structural groups responsible for functionality were identified by analyzing the infrared spectrum obtained in the transmission mode with a Fourier transform infrared spectrometer (FTIR) (FTIR-8400S, Shimadzu).

### Drug loading percentage analysis

DDMSNPs were dispersed in phosphate buffer saline (PBS) and the drug loading percentage was measured using a UV–Vis spectrophotometer (Shimadzu UV 1800, Japan) at 235 nm and 496 nm using the formula:

$$Drug \ loading\ \% = \frac{{\left[Drug\right]}_{i}-{\left[Drug\right]}_{f}}{{\left[Drug\right]}_{t}} \times 100$$

where the total concentration of Drug (free + encapsulated) in the system, ${\left[Drug\right]}_{t}$, and that in the filtrate, ${\left[Drug\right]}_{f}$.

### Drug release study

The drug release study of DDMSNP was performed by suspending the DIM-DOX loaded MSNPs in 10 ml of phosphate buffer saline (PBS) of pH 7.4 in triplicate and shaken at 37℃ in dark condition. At certain intervals of time, the supernatant was removed and recorded the absorbance at 235 nm and 496 nm for DIM and DOX solutions, respectively. The percent release of the drugs was calculated using the following equation:

$$\% \ Drug\ release = \frac{{\left[Drug\right]}_{t}}{{\left[Drug\right]}_{\theta }} \times 100$$

where [Drug]_t_ is the total concentration of the drug and ${[{\text{Drug}}]}_{\uptheta }$ is the concentration of the drug measured at every hour.

### In vitro study

#### Cell culture and reagents

Human breast cancer cell line MDAMB-231, mouse mammary epithelial cell line 4T1 and murine melanoma cell line B16, were cultured in RPMI-1640 or DMEM (Invitrogen) supplemented with 10% heat inactivated fetal bovine serum (Gibco, USA) and 1% penicillin/streptomycin in 5% CO_2_ at 37 °C.

MCF-10A, breast epithelial cell line was cultured in Dulbecco’s modified Eagle’s medium/Nutrient Mixture F-12 Ham supplemented with 100 ng/ml cholera toxin (Sigma, St. Louis, MO, USA), 10 ng/ml epidermal growth factor (EGF) (Thermo Fisher Scientific, Inc., Waltham, MA, USA), 10 µg/ml insulin, 500 ng/ml hydrocortisone and 5% heat-inactivated horse serum (all from Sigma) [17].

#### Flow cytometric analysis

MDAMB-231 and 4T1 cells were trypsinized and collected in microcentrifuge tubes after being treated with DOX and DDMSNP. The media was then removed by centrifugation and 500 µl of PBS was added. The supernatant was then removed, and 4% paraformaldehyde was added and incubated for 10 min on ice. The cells were then washed with PBS again, and primary antibodies of N-cadherin (Alexa Fluor™ 594 tagged), E-cadherin (PerCP-Cyanine5.5 tagged) were added for 2 h and flow cytometry was carried out with a FACS LSRFortessa X-20.

#### Generation of mammospheres from breast cancer cell lines

MDAMB-231 and 4T1, grown as monolayer were trypsinized and seeded at a density of 1 × 10^5^ cells/ml in serum free media with growth supplements in poly-HEMA coated 6 well plates. Cancer stem cells enriched spherical colonies are known as tumorospheres or mammospheres which was generated within 7 days. These spheres were then subjected to treatment with DOX and DDMSNP for 4 h and used for different experiments like flow cytometry, western blotting etc.

#### Analysis of CSC and EMT markers’ expression by flow cytometry

After DOX and DDMSNP treatments, sphere-forming cells were collected and spinned down to eliminate excess medium. 4% paraformaldehyde was added after the PBS wash and placed on ice for 10 min. Following a PBS wash, the supernatant was discarded and the cells were labelled with fluorochrome-conjugated monoclonal antibodies (BD Biosciences, San Diego, CA, USA) against CD44 (FITC) and CD24 (PE). The expression of different EMT markers, such as N-cadherin (AlexaFluor™ 594 tagged) and E-cadherin (PerCP-Cyanine5.5 tagged), in the CD44^+^/CD24^−/low^ CSC population was examined using the FACS LSRFortessa X-20 and analysed by BD FACSDiva™ Software.

#### Cell sorting

1 × 10^7^ cells from 4T1 spheres, as well as monolayer culture and MDAMB-231 spheres, were incubated with primary antibody against CD24 according to the manufacturer's procedure mentioned in CD24 Microbead Kit (MiltenyiBiotec). Following that, goat anti-mouse IgG microBeads (Miltenyi Biotec) were added to the labeled cells and MiniMACS columns (Miltenyi Biotec) were used to magnetically separate the cells. CD44 microbeads (Miltenyi Biotec) were added to acquire CD24^−^ cells at 4 °C for 15 min. Cells were again washed and magnetically separated. The CD44^+^/CD24^−/low^ CSC population was collected for further experiments.

#### Kaplan–Meier plotter analysis

Kaplan–Meier Plotter (https://kmplot.com/analysis/) was used to study the survival information associated with N-cadherin and E-cadherin in TNBC patients.

#### TNMplot data analysis

TNMplot (https://tnmplot.com/analysis/) was used for analyzing gene expression in various normal, tumor and metastatic tissues. In our present study, expressions of N-cadherin and E-cadherin were compared between normal, tumor and metastatic tissues of breast by using this tool.

#### ROC plot analysis

ROC Plotter (https://www.rocplot.org/) is a user-friendly online tool which was used to assess the expression of genes of interest (N-cadherin and E-cadherin) in the face of DOX treatment received by the TNBC patients.

#### Bioavailability radar model-based prediction

By using canonical SMILES of native DIM and DOX, the bioavailability of these drugs was predicted from six physicochemical properties such as lipophilicity, size, polarity, insolubility, in-saturation and flexibility of radar model.

#### Compound optimization by BOILED-Egg model-based prediction

BOILED-Egg model obtained from SwissADME online server, a plot between wLogP vs Topological polar surface area (TPSA) were used to ascertain the penetration of native drugs viz., DIM and DOX through tight junction (BBB).

#### Bioinformatics based ADME prediction

Several ADME properties such as physicochemical (Mol. weight, TPSA), lipophilicity (iLogP, wLogP), pharmacokinetics (gastrointestinal (GI), skin (LogKp), BBB permeability and P-gp substrate, drug likeliness (lipinski, bioavailability score) of native DIM and DOX were predicted using the SwissADME based online server (http://www.swissadme.ch/).

#### Drug combination analysis

DIM and DOX were added separately and together in a constant ratio, as calculated from a dose–effect curve. The inhibition effect was graded on a scale of 0 to 1, with 0 representing no impact and 1 representing 100% effect. The combination index (CI), fraction affected (Fa) and the dose reduction index (DRI) were calculated using CompuSyn software (version 1.0; T. C. Chou and N. Martin, Memorial Sloan-Kettering Cancer Center, New York), and an isobologram was created to quantify the effect of drug interactions. The same procedure was followed in case of DIM encapsulated in DDMSNP (DDMSNP1) and DOX encapsulated in DDMSNP (DDMSNP2).

#### Cell viability assay

Cell viability assay was performed using MTT [3-(4,5-dimethylthiazol-2-yl)-2,5-diphenyl-tetrazolium bromide] (Sigma-Aldrich, USA). MDAMB-231, 4T1 and MCF-10A cells were cultured in 96-well plate and treated with native DIM, native DOX, DIM-DOX in combination, NDIM, NDOX and DDMSNP for 24 h. Treated cells were then incubated in fresh medium containing MTT (0.5 mg/ml) at 37 °C for 3 h. Following that, DMSO was used to solubilize the formazan crystals and the absorbance was measured at 570 nm using an automated micro-plate reader (Infinite® 200 PRO, TECAN, Switzerland).

#### Immunoblotting

After 4 h treatment of DOX and DDMSNP, MDAMB-231 and 4T1 cells were harvested in lysis buffer and whole cell lysates were prepared by using standard protocol [18]. Each sample was an identical amount of protein lysate (100 µg) for western blot analysis. SDS-PAGE was used to separate proteins, which was then transferred to PVDF membranes. Membranes were blocked by 5% non-fat dry milk. After three washes, the membranes were incubated for 2 h at room temperature with primary antibodies in TBS. After three additional washes, membranes were incubated for 2 h at room temperature with HRP-conjugated secondary antibodies. Proteins of interest were visualized under chemi-luminescence (Invitrogen™ iBright™). EMT induction in TNBC cells was confirmed using antibodies against N-cadherin, E-cadherin, Snail, Slug, Vimentin, β-Actin, α-tubulin etc. (Cell Signaling Technology, Abcam and Santa Cruz Biotechnology).

#### Annexin V/PI apoptosis assay by flow cytometry

After 48 h treatment, MDAMB-231 and 4T1 cells were trypsinized and washed with PBS (pH 7.4). Then the cells were resuspended in 500 µl of binding buffer and washed again with PBS. After that 5 µl of FITC-Annexin V and propidium iodide (PI) were added and incubated for 15 min in dark condition. After incubation 400 µl Annexin-binding buffer was added and passed through nylon mesh and kept in ice and analyzed by flow cytometry. Data were acquired FACS LSRFortessaX-20 and analysed by BD FACSDiva™ Software.

#### Quantitative real time PCR (qRT-PCR)

According to the manufacturer’s instruction total RNA was extracted from DOX and DDMSNP treated MDAMB-231 and 4T1 cells using TRIzol reagent (Invitrogen, NY, USA). For each sample, 2 μg of RNA was converted to cDNA using cDNA isolation kit (Bio-Rad, USA). The samples were then used for qRT-PCR analysis using Power SYBR Green Master Mix on Roche LightCycler® 96 System (Switzerland). Data was analyzed by light cycler 96 module1.1.

The list of primers (human and mouse) used for qRT-PCR analysis:

**Table:** 

H-N-cadherin	F-CCTCCAGAGTTTACTGCCATGAC
	R-GTAGGATCTCCGCCACTGATTC
H-E-cadherin:	F-CCTCCTGAAAAGAGAGTGGAAG
	R-TGGCAGTGTCTCTCCAAATCCG
H-GAPDH:	F-GAAAGCCTGCCGGTGACTAA
	R-TTCCCGTTCTCAGCCTTGAC
M-N-cadherin:	F-CAGCCGGAGAACAGTCTCCAA
	R-GCGAGCTGGTAACAAATAGCG
M-E-cadherin:	F-AACCCAAGCACGTATCAGGG
	R-ACTGCTGGTCAGGATCGTTG
M-GAPDH:	F-GAAGGTCGGTGTGAACGGATTT
	R-ATGAGGTCCACCACCCTGTT

#### EMT induction

EMT was induced in MDAMB-231 and 4T1 cells in standard culture medium (6 ml/10 cm plate) containing StemXVivo® EMT Inducing Media Supplement (1X) (R&D Systems) by following manufacturer’s instruction. Cells were then incubated at 37 °C with 5% CO_2_ for 3 days followed by the replacement of fresh culture media with same supplement. After 5 days cells were collected and subjected to different experiments.

#### Transfection

E-cadherin construct and siRNA against E-cadherin were transfected by using lipofectamine™ 2000 (Thermo Fisher Scientific) according to the manufacturer's instructions. The cells were used for further experiments at 24 h after transfection.

#### Encapsulation of DDMSNP in exosome and loading confirmation by TEM

Initially, B16 cell line was treated with DDMSNP and cultured in DMEM with 10% FBS in 5% CO_2_ at 37 °C. These cells then secreted exosomes loaded with DDMSNP. After 24 h of treatment cells were washed and re-seeded them into a new flask with fresh medium. The cell conditioned medium was collected after 48 h of culture and exosomes were isolated using Total Exosome Isolation kit (Invitrogen, cat. No. 4478359) according to manufacturer's instructions and stored at − 80 °C for further use.

The size and morphology of empty exosomes and DDMSNP encapsulated exosomes (e-DDMSNP) were examined using a JEOL JEM-2100 transmission electron microscope (JEOL, Inc., Peabody, MA, USA) at an acceleration voltage of 300 kV.

#### Confocal microscopy

MDAMB-231 cells were grown on coverslips in a petri dish for 16–20 h and treated with e-DDMSNPs for 4 h. After that, cells were fixed in 4% paraformaldehyde in PBS for 10 min before being rinsed three times with PBS. Permeabilization in 0.5% Triton X-100 for 5 min was followed by 30 min of blocking with 2% BSA in PBS. After that, the slides were then incubated in a humid chamber overnight at 4 °C with exosome marker CD63 (Thermo Fisher Scientific) in PBS and washed three times with 0.3% Triton X-100 in PBS. The cells were then treated with FITC conjugated secondary antibodies (1:250) in PBS containing 0.3% Triton X-100 and 10% normal goat serum for 2–3 h at 37 °C before being rinsed three times with PBS containing 0.3% Triton X-100. Finally, the samples were mounted on clean glass slides with vecta shield mounting medium and stored at 4 °C until examined under a confocal laser scanning microscope (Leica).

After treating with e-DDMSNPs for 4 h, spheres were transferred from a 6-well plate to the well of 12-well plate and washed with PBS and placed in the incubator for 1 h for settle down. After that, it was fixed with 4% paraformaldehyde for 20 min and then washed with 0.25% triton-x. Following 3% BSA blocking, the spheres were washed with PBS. Primary antibody against CD63 was then added and kept at 4 °C overnight. After washing, FITC conjugated secondary antibody was added and then nucleus was stained with DAPI and visualized under confocal microscope.

### RFP-expressing orthotopic breast cancer model

#### Establishment of RFP expressing stable cell line

18 h before transfection, highly invasive cells from 4T1-derived spheres were seeded into 6-well plates (3 × 10^5^ cells/well). RFP expressing vector (addgene) was transfected into the cells of 4T1 derived spheres at a 1:10 ratio using lipofectamine™ 2000 (Thermo Fisher Scientific) according to the manufacturer's instructions. The reporter plasmid was utilized to select G418-resistant cells. After 48 h incubation time, the cells were cultured for four weeks in media supplemented with G418 at 400 μg/ml. The resultant cell clones with stable expression of RFP were selected and cultured to grow CSC enriched spheres for further investigation. These spheres were then subjected to MACS column-based cell sorting to isolate CD44^+^/CD24^−^ RFP expressing CSCs.

#### Experimental animals

From Chittaranjan National Cancer Institute’s animal colony (Kolkata, India) adult BALB/c female mice (6–7 weeks old, 25 g b.w.) were collected and utilized for this study. They were kept under typical environmental conditions (temperature: 23 ± 2 °C, relative humidity: 80%, 55 ± 10% and 12 h/12 h light and dark conditions) and fed a standard pellet diet (EPIC mice pellet from Kalyani Feed Milling Plant, Kalyani, West Bengal, India) with ad libitum. All experimental procedures were carried out in strict accordance with the guidelines for the Purpose of Control and Supervision on Animal Experimentation (CPCSEA) and were approved by the Institutional Animal Ethics Committee (Reg. No.-1774/GO/RBi/S/14/CPCSEA, India). After completion of the experiment, all waste materials were disposed off in a safe and hygienic way.

#### Experimental design

BALB/c mice were used to determine the effect of e-DDMSNP on the correlation of up and down-regulating EMT factors with distant localization of neoplastic cells by RFP-fusion protein expressing CSCs sorted from stable cell line on breast cancer metastasis in a 4T1 orthotopic mouse model. Solid tumor was induced by injecting RFP expressing 4T1 derived CSCs (5 × 10^5^) in 100 μl serum-free solution into the mammary gland (underneath the nipple). Then tumors were allowed to grow for a period of 10 days and mice were distributed into five groups (n = 12). Six animals from each group were kept to check the survivability, while six animals from each group were taken for further studies. The experimental mice were distributed in the following groups-

- RFP expressing 4T1 derived CSCs: RFP expressing 4T1 derived CSCs were implanted into the mammary fatpad to each mouse of this group and left untreated for 10 days. Then each animal was injected regular saline in tumor niche for 10 days.
- RFP expressing 4T1 derived CSCs + exosomes: Each animal in this group was injected with stable 4T1 derived CSCs into the mammary fatpad and kept untreated for 10 days. The treatment was then maintained for 10 days with the empty exosomes (5 mg/kg b.w.) injected in tumor niche.
- RFP expressing 4T1 derived CSCs + MSNPs: After inoculation of stable 4T1 derived CSCs in mammary fatpad of each animal of this group was kept untreated for 10 days. The treatment was thereafter carried out for 10 days with empty MSNPs (5 mg/kg b.w.) injected in tumor niche.
- RFP expressing 4T1 derived CSCs + DOX: Each animal in this group was given stable 4T1 derived CSCs implanted into the mammary fatpad and was kept in untreated condition for 10 days. The treatment was continued for another 10 days with DOX (5 mg/kg b.w.) injected in tumor niche.
- RFP expressing 4T1 derived CSCs + e-DDMSNP: All animals in this group had stable 4T1 derived CSCs inoculated into the mammary fatpad and were kept in untreated condition for 10 days. After that, those animals were treated with e-DDMSNP (5 mg/kg b.w.) injected in tumor niche for 10 days.

The mice were sacrificed at 21st day after stable 4T1 derived CSCs implantation except the mice reserved for survivability study. Furthermore, the lungs were washed in PBS and placed in a 4% formalin solution for 48 h before being dehydrated, embedded, and cryosectioned for H & E and immunofluorescence staining. In average n = 6 number of nodules per group were considered for the results. The organs of RFP-expressing 4T1 derived CSCs generated tumor-bearing mice were subjected to organ imaging also.

### Tumor growth response and survivability study

The antitumor efficacy of DOX and e-DDMSNP was assessed by measuring the inhibition of tumor volume and viable tumor cell count by trypan blue dye exclusion method [19]. The tumor growth response was assessed on the basis of median survival time (MST) and percentage increase in life span (% ILS). Mean survival time (MST) was calculated according to the equation: MST = (day of first death + day of last death)/2. The percentage of increase in life span (ILS) was calculated using the equation: ILS% = (T–C)/C × 100, where T represents MST of treated animals and C represents MST of tumor control group. T/C% (treated vs. tumor control) was calculated as MST of treated animals/MST of tumor control group. TIR% (tumor-growth inhibition rate) = (A–B)/A × 100, where A represents mean tumor volume of tumor control group and B represents mean tumor volume of treated group.

### Mass spectrometry

Existence of e-DDMSNP in solid tumor tissue was confirmed by mass spectrometry [20]. Briefly, tumor tissue was frozen in liquid nitrogen and then crushed using a cooled mortar and pestle. 200 μl of diethyl ether was added to the crushed tissue and sonicated for 5 min on a bath sonicator. After vortexing the sample was centrifuged at 10,000 rpm for 10 min and the supernatant was collected for mass spectrometric analysis. Additionally, native DIM and native DOX were also used as reference for mass spectrometry.

### Organ imaging

Organ imaging was used to monitor cell proliferation and metastasis in RFP expressing 4T1 derived CSCs injected mice of different treatment groups. Tumor tissue and lung were collected for organ imaging and data were acquired by IVIS® Lumina LT In Vivo Imaging System, PerkinElmer.

### Histology & immunofluorescence (IF) assay

Tumor tissue, lung, heart, kidney, liver and spleen were collected from all groups of mice. After 24 h of fixation in 10% formalin, the tissue samples were dehydrated in ascending concentrations of ethanol, cleared in xylene and embedded in paraffin blocks then processed for conventional paraffin-embedded histology with hematoxylin and eosin (H & E) staining. The sections were observed under light microscope (DM 1000, Leica, Germany) and photomicrographs were taken with the software LAS EZ.

For IF, tissue sections from control group and mice treated with DOX or e-DDMSNP were antigen-retrieved using citrate buffer. This was followed by blocking with 3% BSA and an overnight incubation at 4 °C with primary antibodies against BRCA1, N-cadherin, and E-cadherin. After that, the tissue sections were incubated for 2 h at room temperature with fluorochrome-conjugated secondary antibodies, such as AlexaFluor™ 594 and AlexaFluor™ 488. Finally, cell nuclei were stained with DAPI and the sections were examined with a fluorescent microscope (Olympus BX53, Japan).

## Result

### Synthesis and characterization of both DIM and DOX loaded mesoporous silica nanoparticles (MSNPs)

**Fig. 1:**
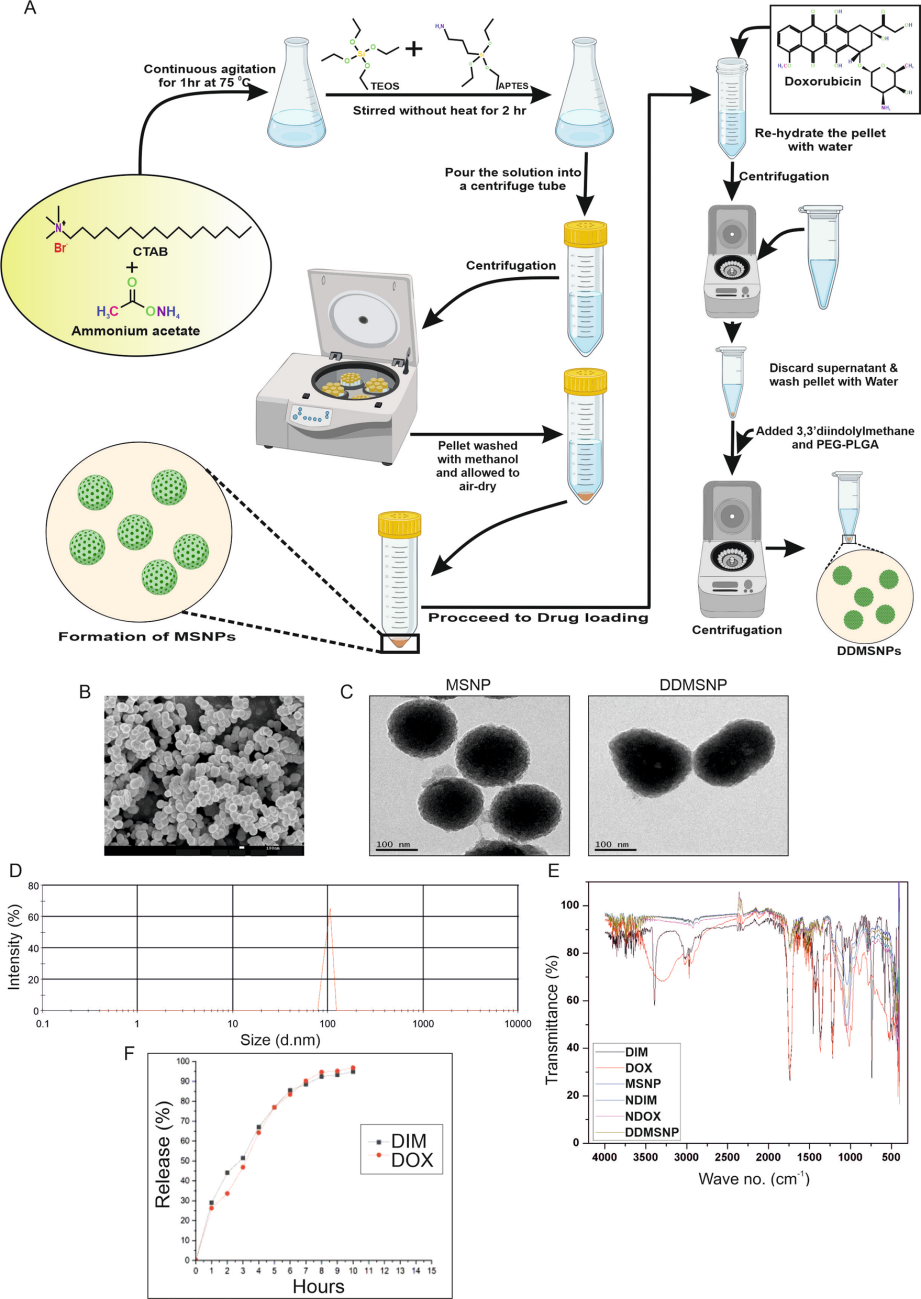
Synthesis and characterization of MSNP and DDMSNP. **A** Modified Stӧber synthesis protocol for the preparation of MSNP and DDMSNP. **B** SEM image shows spherical conformations and ~ 100 nm size of synthesized MSNPs. **C** TEM image represents the morphological analysis of the empty MSNP as well as the loading of DIM and DOX within the MSNP. **D** DLS analysis shows an average size of DDMSNP to be 80–150 nm. **E** FTIR analysis shows comparative spectra of DIM, DOX, MSNP, NDIM, NDOX and DDMSNP to confirm successful encapsulation of DIM and DOX in the nanoparticles. **F** Drug release study of DDMSNP shows a prolonged release of DIM and DOX from the nanoparticle at physiological pH 7.4

DIM and DOX loaded PEG-PLGA based MSNPs were synthesized and characterized by different techniques (Fig. 1). Scanning electron microscopy (SEM) images showed that MSNPs were spherical in shape and homogeneously dispersed (Fig. 1B). Internalization of DIM and DOX into MSNPs was confirmed by Transmission electron microscopy (TEM) (Fig. 1C). Morphological investigation of empty MSNPs and drug loaded MSNPs was also performed by TEM (Fig. 1C). Dynamic light scattering (DLS) was executed to determine the size of DIM and DOX loaded MSNPs (DDMSNPs). Results showed that size distribution of DDMSNPs were ranging from 80 to 150 nm (Fig. 1D). Infrared spectroscopy provided a plethora of knowledge about the functional groups of the nanoparticle, in particular, furnished information about the arrangement and packing of the surface chains. To improve the coverage of shell material on the surfaces of the core particles, bi-functional molecules were frequently used to modify their surfaces. Organic compounds with dual functions, like APTES, had been used to modify the surface of core particles. At one end of this molecule was an ethoxy group; while at the other was an amino group. Through the hydroxyl group, APTES created a covalent link with silica nanoparticles, which caused their surface to become NH-terminated. DIM showed characteristic band in the region of 3394 cm^−1^ and had been shifted to 3420 cm^−1^ in DIM-MSNP (NDIM) which was attributed to NH stretching (Fig. 1E). Further, C–H stretching in DIM was shifted from 2921 cm^−1^ to 2925 cm^−1^ in NDIM. The indole group was identified as the cause of the distinctive band in the region, which was 1641 cm^−1^ in DIM and shifted to 1648 cm^−1^ in NDIM. The aromatic bonding (C=C) was observed from the FTIR studies by measuring shift in peak position from 1540 cm^−1^ to 1543 cm^−1^. Hence, these shifts in peak position were due to the interaction between the DIM and MSNP. FTIR spectrum of free DOX revealed unique peaks at wave numbers: 668 cm^−1^ corresponding to Alkyne C–H bonds, between 1022 cm^−1^ and 1096 cm^−1^ corresponding to aromatic C–H bonds, 1406 cm^−1^ corresponding to carboxylic acids, 1636 cm^−1^ corresponding to Alkenyl C=C stretching, 2086 cm^−1^ corresponding to amino group, 2352 cm^−1^ corresponding to charged amines C=NH^+^, 3453 cm^−1^ corresponding to O–H stretching (Fig. 1E). Peak shifting of these bonds were observed in Fig. 1E for DOX loaded MSNP (NDOX). The FTIR peak at around 466 cm^–1^ showed Si–O–Si bending mode and that at in the range of 750–850 cm^−1^ corresponding to NH_2_ for the amino functionalized MSNP. Primary, secondary and tertiary amines showed peaks in different wavenumbers. The primary amines showed two peaks, one with a broader peak at 3440 cm^–1^ and one minor peak around 1631 cm^–1^ associated with NH_2_ asymmetric stretching. The NH and NH_2_ peaks indicated the functionalization of silica surface with amine groups (Fig. 1E). In case of DIM loaded MSNPs, the strong and wide peak at 3414 cm^−1^ corresponded to the overlapping of hydroxyl and amine groups (from the indolyl group of free DIM) was observed (Fig. 1E). Results also showed characteristic peaks assigned to symmetric (492 and 803 cm^−1^) and asymmetric (1098 cm^−1^) Si–O–Si stretching, and stretching vibrations of Si–OH (941 cm^−1^) for non-functionalized MSNPs (Fig. 1E). The spectrum showed in Fig. 1E corresponded to PEG-PLGA encapsulated DIM and DOX loaded MSNPs or DDMSNPs. The smooth broad band around 3500 cm^−1^ was related to the ability of oxygen to form hydrogen bonds. A small doublet around 3000 cm^−1^ was associated with CH, CH_2_ and CH_3_ groups. The typical peak at 1752 cm^−1^ was due to C=O stretching. Although the characteristic band width at 1182 cm^−1^ could be attributed to the ether group. The characteristic bands at 1130 cm^−1^ and 1452 cm^−1^ were due to the C–O–C group and the CH bond of the methyl group, respectively. As the spectra showed, the characteristic groups of PLGA were stronger than those of PEG, because the first constitutes more than 90% of the block copolymer. Therefore, the presence of PEG was barely detectable by the small signal of C–C–O harmonics located around 950 and 840 cm^−1^. After suspending DIM and DOX loaded mesoporous silica nanoparticles (DDMSNPs) in PBS (pH 7.4), and measuring the absorbance of DIM and DOX in the free and encapsulated form in the filtrate, we calculated the estimated amount of drugs encapsulated in the DDMSNPs to be ~ 84% of DIM and ~ 81% of DOX. Drug release assay of DDMSNPs was performed in PBS (pH 7.4). Results were presented as a percentage of cumulative release over 10 h. A faster release of DIM and DOX was observed at pH 7.4 in a PBS medium. We showed that 50% drug was released after 3 h, whereas, 90% of the drug was released in 8 h in PBS medium (Fig. 1F). This release behavior indicated that the drugs would be released at biological pH. Controlled releases of DIM and DOX were observed from the mesoporous silica nanoplatforms.

### Doxorubicin induces CSC mediated EMT progression

**Fig. 2:**
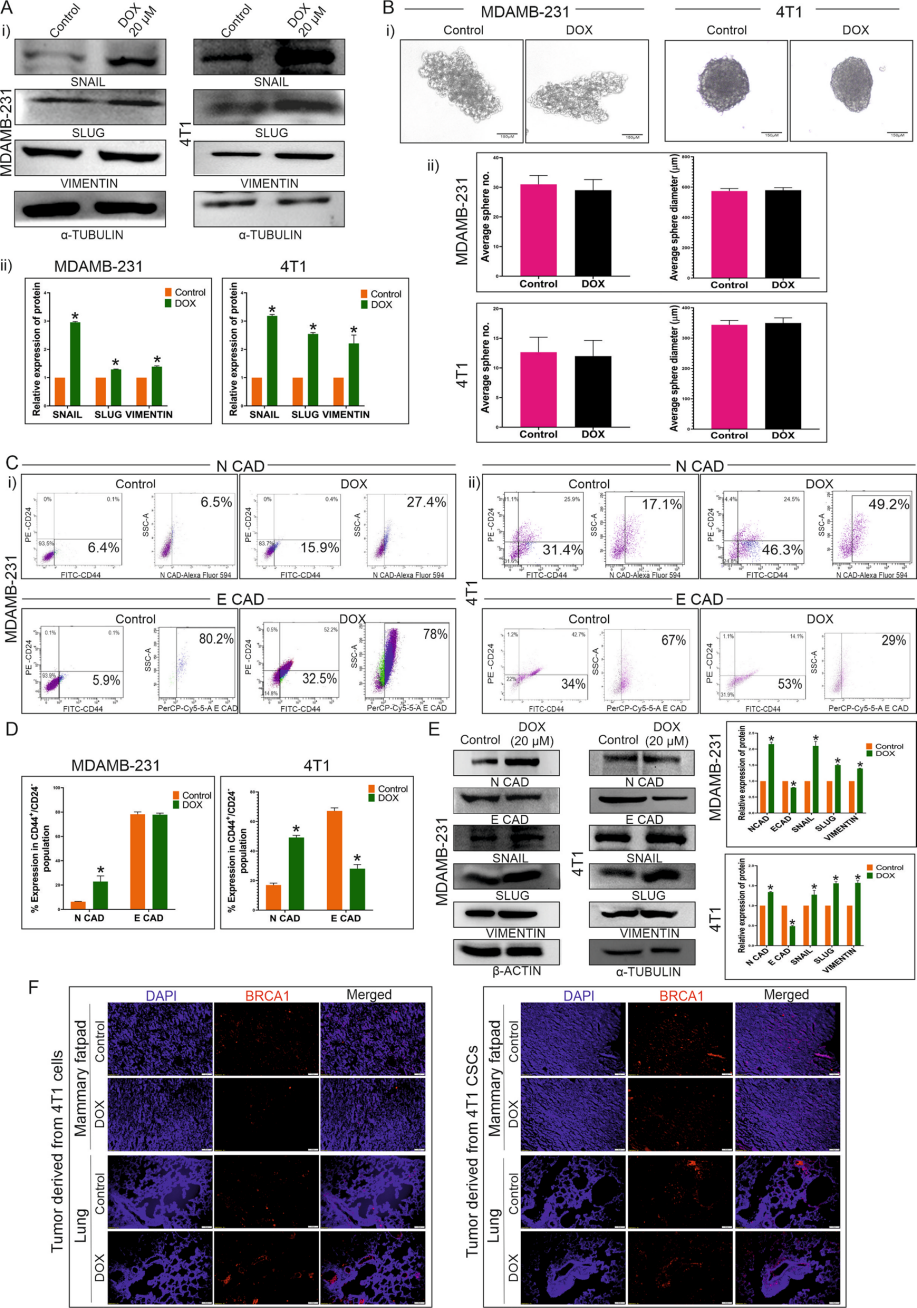
Effect of DOX on TNBC cells and CSCs derived from MDAMB-231 and 4T1 cell lines. **A** Overexpression of Snail, Slug and Vimentin by DOX treatment. (i) Figure shows expression of Snail, Slug and Vimentin in control and 20 µM DOX treated groups of MDAMB-231 and 4T1 cells by western blot analysis. (ii) Graphical illustration denotes mean ± SD, n = 3. * indicates P ≤ 0.0002. **B** (i) Figure represents the sizes of CESs derived from MDAMB-231 and 4T1 cell line after DOX treatment compared to the control. (ii) Graphical representation of diameter and number of CESs of control and DOX treated groups shows no significant differences. **C** (i, ii) FACS analysis evaluates the expressions of N-cadherin and E-cadherin in CD44 ^+ ^/CD^24-/low^ CSC population in both cell line derived spheres of both control and 4 h DOX treated groups. **D** Bar diagram represents significant difference between N-cadherin expression of control and DOX treated groups, * indicates (P < 0.0001) and nonsignificant difference between E-cadherin expression of the same groups in MDAMB-231 derived CESs (left panel). The right panel denotes significant differences between N-cadherin and E-cadherin expressions of 4T1 derived CESs, * indicates (P < 0.0001). **E** Western blot data represents expressions of N-cadherin, E-cadherin, Snail, Slug and Vimentin in DOX treated CESs derived from MDAMB-231 and 4T1 cells. Graphical representation shows the significant differences between the expressions of these proteins in control and DOX treated groups, * indicates (P < 0.01). **F** Immunofluorescence study assesses BRCA1 (red) expression in mammary fatpad and metastatic lung in DOX treated and untreated groups of 4T1 cells-derived tumor-bearing BALB/c mice (n = 6 in each group) (left panel) and 4T1 CSCs- derived tumor-bearing BALB/c mice (n = 6 in each group) (right panel)

DOX was treated to MDAMB-231 and 4T1 cells in a dose-dependent manner, resulting in alteration in the expression of EMT marker such as N-cadherin. At 20 µM dosage, a significant (P < 0.005) change in the expression of this marker was observed in both cell lines, which was consistent at higher dosage as well (Fig. S1Ai, ii). Hence, further experiments were conducted with the same dosage of DOX. Although, expression of E-cadherin was decreased upon DOX treatment to 4T1 cells, no detectable protein expression was observed in case of MDAMB-231 cells. It was quite similar to the earlier reports stating that MDAMB-231 cells have very low E-cadherin expression due to promoter methylation, apparently too weak to be detected by western blot, but can be detected as a small subpopulation in flow cytometry [21]. As the expression of Snail, Slug and Vimentin were increased upon DOX treatment, it is confirmed that DOX promotes EMT in MDAMB-231 and 4T1 cells (Fig. 2Ai, ii). Additionally, number and diameter of CSC enriched spheres (CESs) were not changed after DOX treatment compared to the control spheres (Fig. 2Bi, ii). Flow cytometric analysis revealed that upon DOX treatment percentage of N-cadherin expressing cells were significantly (P < 0.005) increased to 65.7% and 12.6% compared to the control (55.4% and 5.5%) in MDAMB-231 and 4T1 respectively (Fig. S1Bi–iii). On the other hand, a subpopulation of MDAMB-231 cells had shown weak expression of E-cadherin which was not significantly altered when treated with DOX (Fig. S1Bi–iii). Additionally, no significant change upon DOX treatment in the percentage of E-cadherin positive cells was observed in case of 4T1 also (Fig. S1Bii, iii). Surprisingly, noticeable increase in E-cadherin expression was observed in MDAMB-231 derived CESs compared to the MDAMB-231 cells (Fig. S1C.i-ii). Regarding the CESs, DOX caused an increase in the population of N-cadherin expressing cells and a decrease in the population of E-cadherin expressing cells, indicating the onset of EMT as a result of this treatment (Fig. 2Ci, ii, D). However, decrease in E-cadherin positive population upon DOX treatment was not so significant in case of MDAMB-231 derived CESs. In immunoblotting results, similar expressional changes of N-cadherin and E-cadherin levels were observed in the sorted CSCs from both the cells-derived CESs treated with DOX (Fig. 2E). However, increase in the expression of other EMT markers like Snail, Slug and Vimentin were also documented by immunoblotting analysis, which suggests induction of EMT in CSCs by DOX (Fig. 2E). Furthermore, Kaplan–Meier plots derived from patient database supports the fact that high N-cadherin and low E-cadherin expression is associated with high mortality rate in TNBC patients (Fig. S2A). Albeit, according to the TNMplot significant increase in N-cadherin expression was noticed in metastatic TNBC samples compared to the control and non-metastatic tumor group (Fig. S2B). However, there were no discernible differences in the expression of E-cadherin between the normal individuals and the groups of tumor-bearing and metastatic TNBC patients (Fig. S2B). Nevertheless, it is evident from the patient data that, in contrast to the responder group, non-responders to DOX presented higher expression of N-cadherin and lower expression of E-cadherin (Fig. S2C). This suggests that resistance to DOX is not only related to the EMT markers like N-cadherin and E-cadherin, but also contributes to cancer virulence through treatment failure and increased metastasis. This was further confirmed by comparing BRCA1 expression in 4T1 cells with CSCs-derived control and DOX treated metastatic tumors, as well as between lungs of tumor-bearing mice of these groups (Fig. 2F). BRCA1 is a breast cancer marker prominently expresses in 4T1 cells [22]. However, it was intriguing to find out that overexpression of BRCA1 could be observed in lungs of 4T1 derived tumor-bearing mice which suggests distant metastasis of the neoplastic cells. The rate of metastasis was even higher in tumors originated by 4T1-derived CSCs, as evidenced by increased BRCA1 expression in the lungs of those mice groups (Fig. 2F). Nevertheless, DOX significantly increased BRCA1 expression in both 4T1 cells and CSCs-derived metastatic lungs compared to the control group (Fig. 2F). These results suggest that CSCs exposed to DOX were able to induce EMT and metastasis more significantly than the unexposed cancer cells, DOX exposed cancer cells and unexposed CSCs. As BRCA1 is a known activator of tumor suppressor genes, in orthotopic tumor region of mammary fatpad of both 4T1 cells and CSCs injected mice, expression of BRCA1 was reduced in DOX treated groups compared to the controls (Fig. 2F) [23]. These results further confirmed the pro-metastatic and EMT inducing potential of DOX in case of triple negative breast cancer.

### DIM and DOX synergistically inhibited viability of TNBC cells

**Fig. 3:**
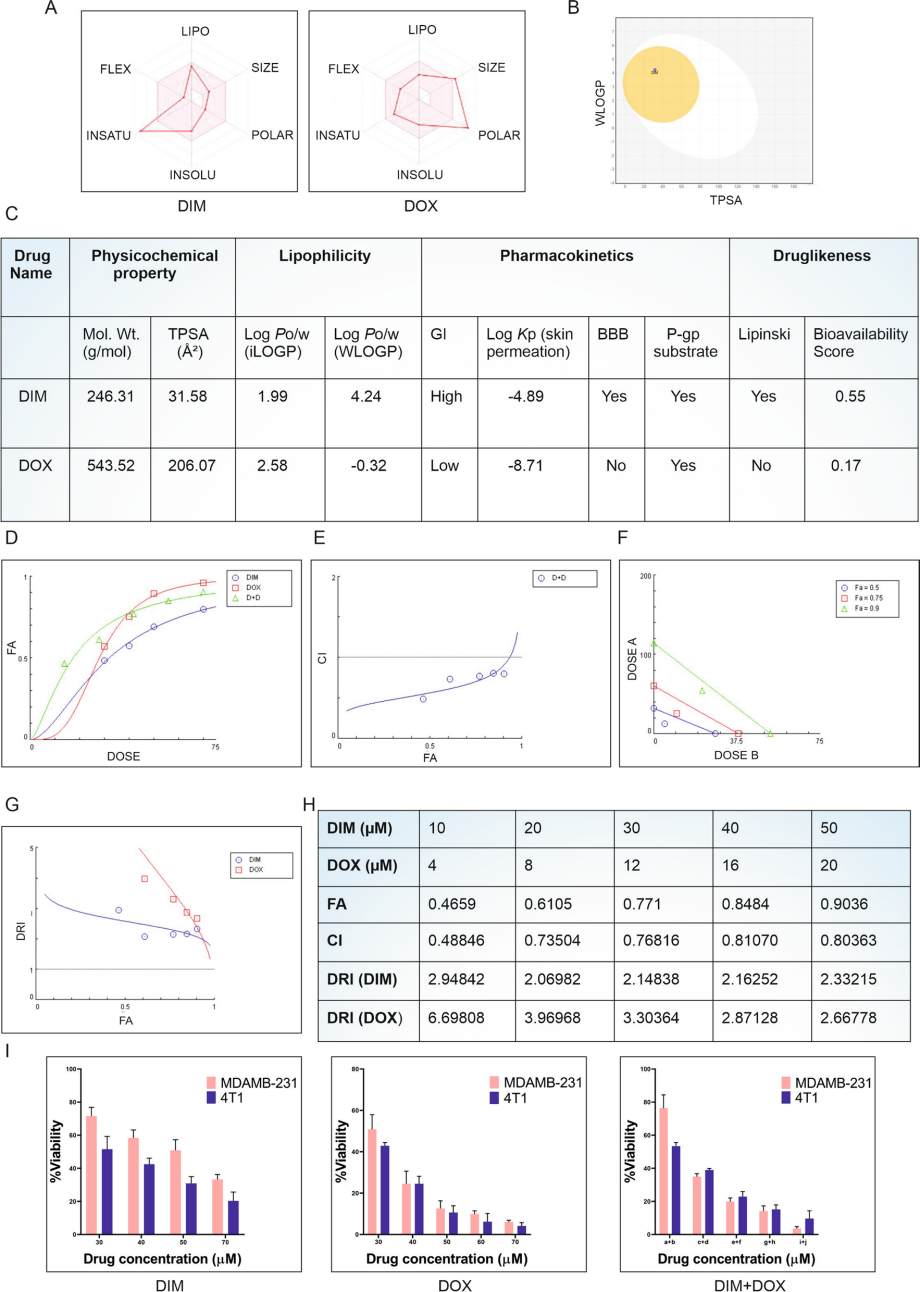
Synergistic effect of DIM and DOX. **A** Bioavailability radar model, **B** boiled-egg model and **C** SwissADME properties of native DIM and DOX is depicted in the figure. CompuSyn analysis of DIM and DOX represents. **D** Dose effect plot of DIM, DOX and D + D (DIM + DOX), **E** plots with CI values of < 1 indicating synergism between D + D (DIM + DOX), **F** isobologram representing effective doses required for inhibition at 50% (Fa 0.5), 75% (Fa 0.75) and 90% (Fa 0.9) for each individual drug. Synergism is demonstrated by the dose pair plotted as a point (symbol) below their respective Fa isobole or line. **G** DRI of the drug combination of DIM and DOX is presented and DRI value > 1 indicates favourable drug combination. **H** All data are the representation of three independent experimental repeats. Fa, fraction affected; CI, combination index; DRI, dose reduction index. **I** MTT data demonstrates individual and the combinatorial effects of DIM and DOX in both the MDAMB-231 and 4T1 cell lines after 24 h of treatment. In this figure, a + b = DIM 10 µM + DOX 4 µM, c + d = DIM 20 µM + DOX 8 µM, e + f = DIM 30 µM + DOX 12 µM, g + h = DIM 40 µM + DOX 16 µM and i + j = DIM 50 µM + DOX 20 µM

DIM is a heterocyclic and bioactive compound which represents potentially appealing anti-cancer agent. The synergistic effect of DIM and DOX on EMT induction, metastasis, and apoptosis in TNBC, on the other hand, is unclear, which could be useful for future therapeutic development. Different bioinformatics-based pharmacokinetics studies were conducted to determine the bioavailability of DIM and DOX. The radar model revealed that DIM has high insaturation and poor solubility, whereas, DOX is a polar compound with high molecular weight and poor bioavailability (Fig. 3A). Furthermore, the pharmacokinetic properties of DIM and DOX were also predicted using the BOILED-Egg model. It was justified from this model that DIM has the ability to absorb by the passive gastrointestinal absorption (HIA), whereas, DOX shows poor absorption potential by gastrointestinal tract (Fig. 3B). Aside from that, the BOILED-Egg plot indicated inadequate DOX penetration through the tight junction like blood brain barrier (BBB) (Fig. 3B). DIM, however, appears to have the ability to pass through BBB in this scenario. Furthermore, SwissADME-based properties indicate the physicochemical properties, lipophilicity, pharmacokinetics and drug likeness of DIM and DOX. While molecules with the polar surface area or TPSA < 60 Å^2^ would be efficiently absorbed (> 90%), those with a TPSA > 140 Å^2^ and beyond would be poorly absorbed (< 10% fractional absorption). From Fig. 3C it was confirmed that DIM could be efficiently absorbed through the cell membrane, whereas DOX might not pass through the same. For the prediction of potential therapeutic ligands, the ADME analyses of drugs were significantly more important. The SwissADME tool was applied to predict ADME properties using the widely utilized Lipinski rule of five (RO5) (i.e., appropriate absorption is more likely when MW < 500 Da, AlogP < 5, Hdon < 5, Hacc < 10) strategies. According to this rule DIM fulfilled the criteria but DOX had 3 violations and bioavailability of DIM was higher than DOX (Fig. 3C). Concentrations of 10, 20, 30, 40 and 50 µM of DIM and 4, 8, 12, 16 and 20 µM of DOX were used to generate a dose–effect curve. A constant ratio (10/4 = 5:2) was used to establish the dosages used in combinatory treatment groups (10 + 4, 20 + 8, 30 + 12, 40 + 16, and 50 + 20 µM). DIM and DOX presented a synergistic effect (Fig. 3D–H). The dose–response effects of DIM, DOX and DIM + DOX were presented in Fig. 3D. CI values (< 1), a quantitative definition for synergism, were found in Fig. 3E, thus indicating a favourable drug combination which may exert synergistic inhibition of cell viability. Isobolograms, representing equipotent combinations of two drugs administered at different dosages, were also established via CompuSyn analysis. The dosages of drug combinations revealed by the isobologram also suggested that DIM and DOX exhibited synergistic effects (Fig. 3F). Dose reduction index (DRI) values were revealed to be > 1 of each drug in combination treatment which indicates a favourable drug combination (Fig. 3G). This measured the number of folds by which single drug dosage could be reduced when used in combination. Data obtained via CompuSyn analysis were presented in Fig. 3H. Figure 3I showed that the combinatorial effect of DIM and DOX was more efficient than the individual dosages of DIM and DOX in both MDAMB-231 and 4T1 cell line. The IC50 value was reduced to 21 µM for DIM and 8 µM for DOX in combinatorial therapy in MDAMB-231 compared to the individual IC50 value of 48 µM and 22 µM for DIM and DOX respectively. On the other hand, in 4T1 IC50 values of DIM and DOX were 17 µM and 7 µM for combinatorial therapy which was much less than individual IC50 i.e., 30 µM and 16 µM for DIM and DOX respectively. Henceforth, these IC50 dosages for the combinatorial treatment with native compounds were used for in vitro investigations.

### Combinatorial effect of DIM and DOX on EMT markers and cell survival

**Fig. 4:**
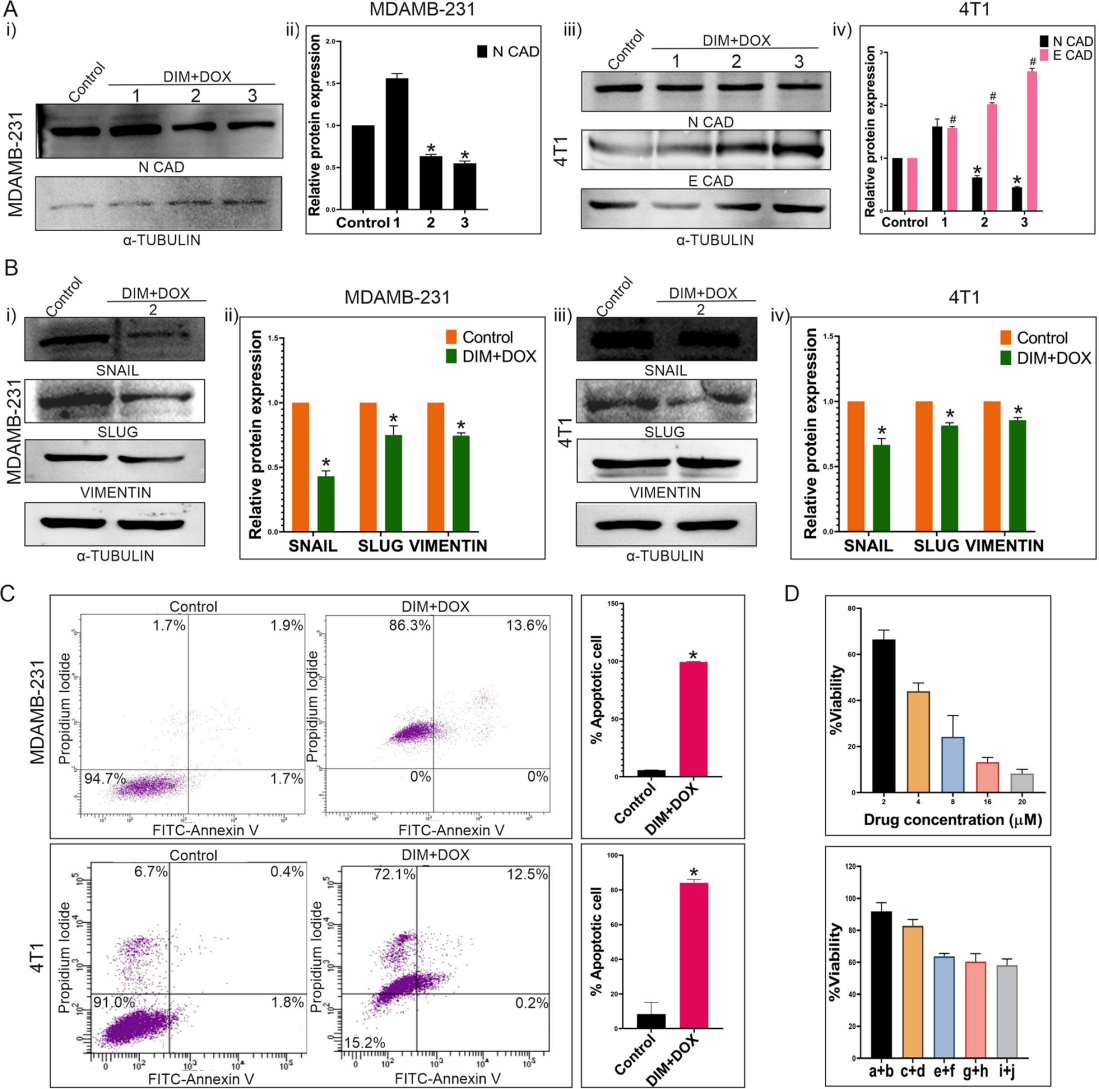
Effect of DIM and DOX combination on EMT induction or apoptosis of TNBC. **A** (i) Expression of N-cadherin upon combinatorial treatment compared with the control. Here, “1” = DIM 10 µM + DOX 4 µM, “2” = DIM 21 µM + DOX 8 µM and “3” = DIM 30 µM + DOX 12 µM. (ii) Graphical representation shows significant change of N-cadherin expression in DIM and DOX treated MDAMB-231 cells compared to the control, * denotes P < 0.05. (iii) Immunoblotting shows the expressions of N-cadherin and E-cadherin. α-Tubulin is considered as the loading control. (iv) Bar graphs depict significant differences of N-cadherin and E-cadherin expressions between control and other groups in 4T1 cells (P < 0.001). Here, “1” = DIM 10 µM + DOX 4 µM, “2” = DIM 17 µM + DOX 7 µM and “3” = DIM 30 µM + DOX 12 µM. **B** (i, iii) Blots shown are the representative of expression of Snail, Slug and Vimentin in DOX treated MDAMB-231 and 4T1 cells compared to the control. α-Tubulin is used as a loading control. (ii, iv) Graphical representation illustrates significant changes in the expressions of Snail, Slug and Vimentin compared to the control (P < 0.002). **C** FACS analysis evaluates apoptotic cell population after 48 h of treatment with DIM and DOX in combination to MDAMB-231 and 4T1 cells. Left most panel shows control plot of untreated cells. This experiment is represented graphically in the rightmost panel (P < 0.0001). **D** Figure depicts the percentage of viable cells after 24 h of treatment with DOX or DIM and DOX in combination to MCF 10A cells. Here, a + b = DIM 30 µM + DOX 4 µM, c + d = DIM 40 µM + DOX 8 µM, e + f = DIM 50 µM + DOX 12 µM, g + h = DIM 60 µM + DOX 16 µM and i + j = DIM 70 µM + DOX 20 µM

Western blotting was used to identify the combination dose of DIM and DOX at which the expressions of EMT markers were altered in MDAMB-231 and 4T1 cells in an attempt to decrease the harmful effects of DOX (Fig. 4A). It was found that when DIM and DOX were treated together at IC50 dosages, N-cadherin expression dramatically decreased (P < 0.05) (Fig. 4A). Treatment with an increased dosage of the same combination resulted in a continuous decrease in N-cadherin expression (Fig. 4A). On the other hand, in contrast to N-cadherin, E-cadherin expression was elevated in 4T1 cells (Fig. 4Aiii, iv). In addition to these, combinatorial treatment of DIM and DOX at IC50 dosage for 4 h in both the cell lines resulted in lower expressions of other EMT markers such as Snail, Slug, and Vimentin (Fig. 4B). However, at a later timepoint of 48 h, the same dosage of this combination triggered apoptosis in both the cell lines, as most of the cells exhibited annexin V-FITC/PI positive when flow cytometric analysis was performed (Fig. 4C). Interestingly, in the instance of the MCF-10A normal breast cell line, the same dosage of DIM and DOX combination failed to reach the IC50 concentration achieved by individual DOX treatment (IC50 = 6.9 µM) with the same dosages as in combination (Fig. 4D). Hence, this is proved that combination of DIM and DOX can restrict EMT progression in TNBC at a significantly low dose and this combination does not affect viability of normal breast cells.

### Mitigation of CSCs mediated EMT of TNBC cells by DDMSNP

**Fig. 5:**
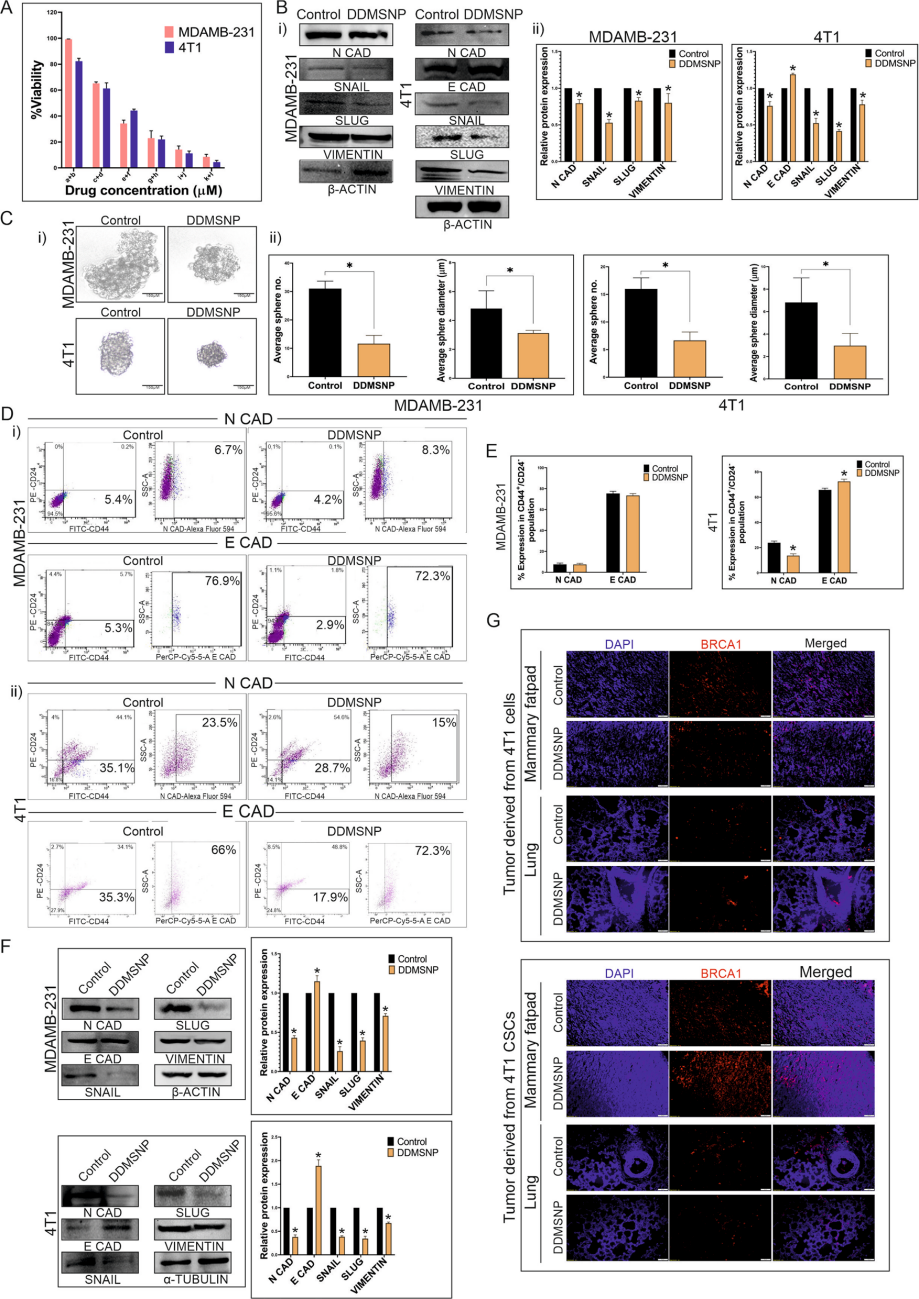
Effect of DDMSNP on TNBC cells and CSCs involved in EMT. **A** Bar diagram represents MTT data showing the percentages of viable cells after 24 h of DDMSNP treatment encapsulating a + b = DIM 0.13 µM + DOX 0.05 µM, c + d = DIM 0.25 µM + DOX 0.1 µM, e + f = DIM 0.5 µM + DOX 0.2 µM, g + h = DIM 1 µM + DOX 0.4 µM, i + j = DIM 2 µM + DOX 0.8 µM and k + l = DIM 4 µM + DOX 1.6 µM. **B** (i) Immunoblotting analysis of DDMSNP (DIM 0.77 µM + DOX 0.31 µM) treated MDAMB-231 and DDMSNP (DIM 0.57 µM + DOX 0.23 µM) treated 4T1 cells shows expression of the proteins involved in EMT. (ii) Graphical representation shows significant changes in the expressions of N-cadherin, Snail, Slug and Vimentin (P < 0.05) in MDAMB-231 and E-cadherin along with the same proteins (P < 0.05) in 4T1 upon DDMSNP treatment. **C** (i, ii) Figures compare the number and diameter of CESs treated with DDMSNP to the control group (P < 0.01). **D** FACS analysis for the expressions of N-cadherin and E-cadherin in CD44 + /CD24-/low CSC population in DDMSNP treated group compared to the control in CSCs derived from MDAMB-231 and 4T1 derived CESs. **E** Graphical illustration denotes change in expression of EMT markers in CSCs derived from MDAMB-231 and 4T1 derived CESs. Figure shows no significant change of the markers in MDAMB-231 CSCs and significant change in 4T1 CSCs (P < 0.0001). **F** Western blot (left panel) and bar diagram (right panel) analysis represents the comparison between expressions of N-cadherin, E-cadherin, Snail, Slug and Vimentin in DDMSNP treated and untreated groups of CSCs from MDAMB-231 (P < 0.01) and 4T1 (P < 0.001) derived CESs. **G** Identification of BRCA1 (red) expression in mammary fatpad and in the metastatic lung of BALB/c mice injected with both 4T1 cells and CSCs by immunofluorescence study

**Fig. 6:**
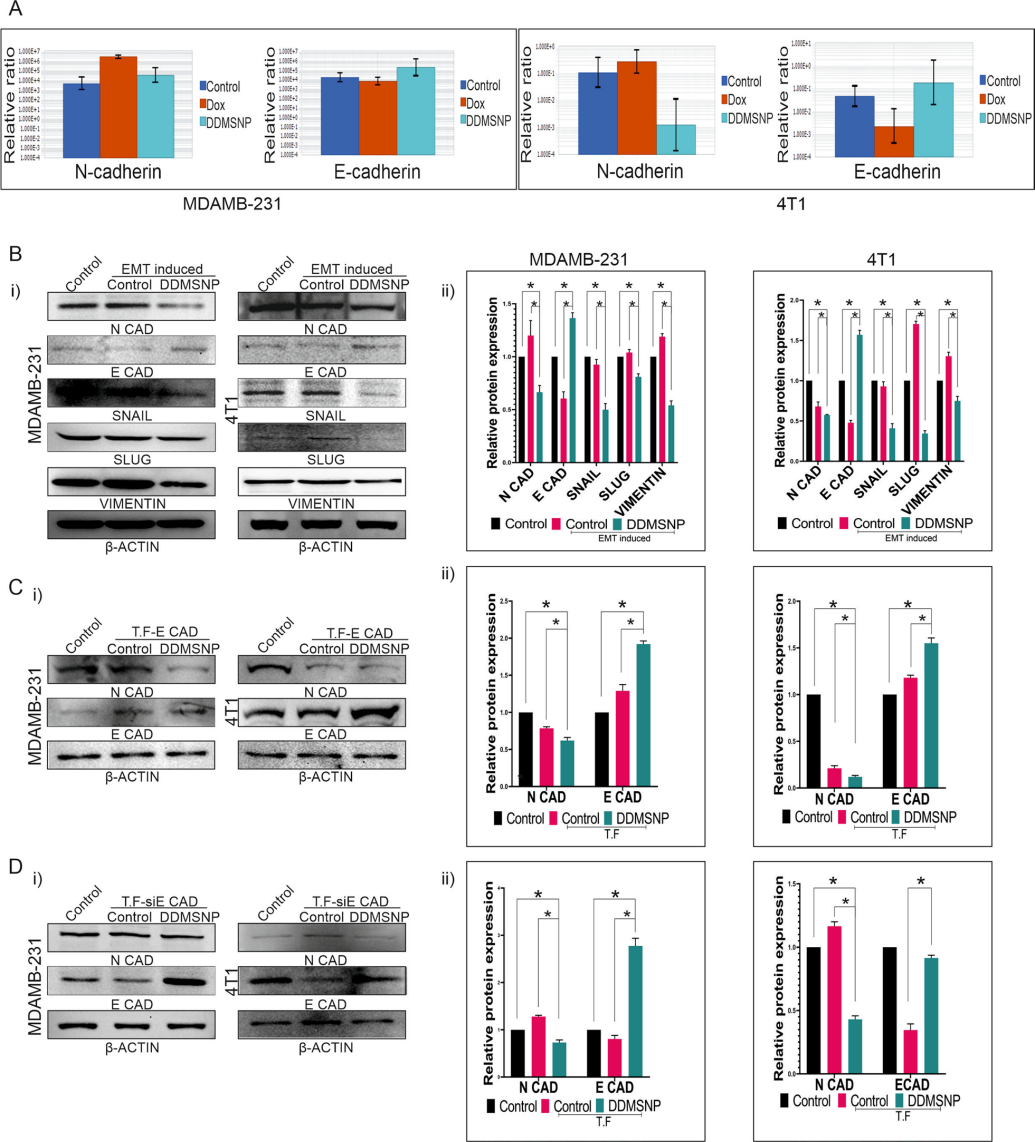
Regulation of in vitro alteration of EMT markers by DDMSNP. **A** Total RNA extracted from DOX and DDMSNP treated and untreated MDAMB-231 and 4T1 derived CSCs are analyzed by qRT-PCR for N-cadherin and E-cadherin. **B** (i) Western blot data depicts the differences between the expressions of N-cadherin, E-cadherin, Snail, Slug and Vimentin in presence of DDMSNP compared to the untreated EMT induced or uninduced CSCs. β-actin is used as a loading control. (ii) The bar diagrams show relative protein expressions. * denotes P < 0.05. **C** (i) Western blot data from CSCs of MDAMB-231 (left panel) and 4T1 (right panel) transfected with E-cadherin expressing vectors shows differential expressions of N-cadherin and E-cadherin in non-transfected control, transfected control and DDMSNP treated transfected CSCs. (ii) Densitometric comparison for N-cadherin and E-cadherin expression among non-transfected control, transfected control and DDMSNP treated transfected CSCs is depicted by bar graphs. * denotes P < 0.05. **D** (i) The expressions of N-cadherin and E-cadherin in non-transfected control CSCs, transfected control CSCs and DDMSNP treated siE Cadherin (siE CAD) transfected CSCs are represented in Western blot data. (ii) Bar graphs show the densitometric comparison of N- and E-cadherin expression in non-transfected control, siE CAD transfected control, and siE CAD transfected CSCs treated with DDMSNP. * denotes P < 0.05

Next, we used the nanotized forms of these compounds in order to increase their effectiveness and to prove their synergistic effect. A dose effect curve was established using concentrations of 0.25, 0.5, 1, 2, and 4 µM of nanotized DIM (NDIM) and 0.1, 0.2, 0.4, 0.8, and 1.6 µM of nanotized DOX (NDOX). The dosages utilized in the combinatory treatment groups (0.25 + 0.1, 0.5 + 0.2, 1 + 0.4, 2 + 0.8, and 4 + 1.6 µM) were determined using a constant ratio (0.25/0.1 = 2.5:1). According to Fig. S3A-E, NDIM and NDOX produced a synergistic impact. The effects of NDIM, NDOX, and both DIM & DOX loaded MSNP (DDMSNP) on dose response were displayed in Fig. S3A. A favourable combination of drugs that may exhibit synergistic inhibition of cell viability was observed in Fig. S3B, having CI values (< 1), a quantitative definition for synergism. The dosages of drug combinations revealed by the isobologram also suggested that DIM and DOX in its nanotized form (DDMSNP) exhibited synergistic effects (Fig. S3C). It was discovered that the dose reduction index (DRI) values for each drug in the combination treatment were > 1, indicating a beneficial drug combination (Fig. S3D). This determined how many times the dosages of individual drugs may be decreased when taken together. CompuSyn analysis generated data showed that native DIM and DOX, nanotized DIM and DOX in form of DDMSNP also showed synergistic activity during treatment (Fig. S3E). From the data we observed that, the effect of NDIM and NDOX was more prominent than the individual dosages of the native form of these drugs against both the cell lines MDAMB-231 and 4T1 (Fig. S3F). However, when treated with DDMSNP to MDAMB-231 cells, the IC50 values for DIM and DOX were decreased to 0.77 µM and 0.31 µM, respectively, from their individual IC50 values of 5.7 µM and 1.4 µM (Fig. 5A, Fig. S3F). In contrast, the IC50 values of DIM and DOX in 4T1 were 0.57 µM and 0.23 µM for DDMSNP treatment, which were significantly lower than the individual IC50 values of 4.14 µM and 0.96 µM for NDIM and NDOX respectively (Fig. 5A, Fig. S3F). Hence after, these IC50 concentrations for combinatorial treatment with native drugs were eventually employed for in vitro studies. Following that, immunoblotting was performed to examine the regulation of EMT markers in both cell lines upon DDMSNP treatment and the reduction of the expressions of N-cadherin, Snail, Slug and Vimentin were observed and the expression of E-cadherin in 4T1 was raised compared to the control (Fig. 5Bi, ii). Furthermore, there was a significant reduction in the number and diameter of the DDMSNP treated CESs in compared to control (Fig. 5Ci, ii), which confirmed opposite effect of DOX as it maintained CESs propagation (Fig. 2Bi, ii). However, DDMSNP treatment to the cancer cells caused significant (P < 0.005) reduction in N-cadherin expression of MDAMB-231 cells and notable increase in E-cadherin expression of 4T1 cells (Fig. S4A–C). No significant changes in the expressions of E-cadherin in MDAMB-231 and N-cadherin in 4T1 cells were observed (Fig. S4A–C). Henceforth, to verify the regulation of EMT markers upon DDMSNP treatment, FACS analysis was performed. It was observed that in CD44^+^/CD24^−/low^ population, unlike DOX treated groups, neither N-cadherin nor E-cadherin showed any significant changes in DDMSNP treated MDAMB-231 derived CSCs, which suggested restricted EMT progression (Fig. 5Di, E). In case of 4T1 derived CSCs, upon DDMSNP treatment the expression of N-cadherin decreased from 23.5 to 15% and E-cadherin was increased from 66 to 72.3%, which also suggested regulation of EMT (Fig. 5Dii, E). This was further confirmed in sorted CSCs from MDAMB-231 and 4T1 derived CESs by immunoblotting analysis. It was observed that when CSCs were treated with DDMSNP, the expression of E-cadherin increased while the expression of N-cadherin, Snail, Slug, and Vimentin decreased (Fig. 5F). Further, post-EMT metastasis to lungs of mice injected with either TNBC cells (4T1) or CSCs derived from 4T1 cells were compared by immunofluorescence intensity of BRCA1 marker. From the results it was confirmed that compared to the 4T1 cells-injected groups, CSCs-injected groups showed more metastasis to lungs of tumor-bearing mice, which was denoted by increased BRCA1 expression (Fig. 5G). But it was evident from the immunofluorescence analysis of BRCA1 that DDMSNP was able to reduce post-EMT metastasis of tumor cells to lungs in both 4T1 and CSCs-injected tumor-bearing mice (Fig. 5G), which was just opposite to our previous result where we found DOX induced post-EMT metastasis (Fig. 2F). Along with these, expressions of N-cadherin and E-cadherin in mRNA level were also checked by quantitative RT-PCR (Fig. 6A). Upon DOX treatment N-cadherin expression was increased and E-cadherin expression was decreased, which was reversed when treated with DDMSNP to MDAMB-231 derived CSCs (Fig. 6A). The same outcomes were noted for CSCs generated from 4T1 (Fig. 6A). Efficacy of DDMSNP was further proved in CSC-enriched CESs exposed or unexposed to EMT inducing media. The result was contradictory to the effect of DOX on N-cadherin overexpression and decreased E-cadherin expression in CSCs generated from TNBC cells (Fig. 2E). The MDAMB-231 and 4T1 derived CSCs exposed to EMT induction showed lower expression of N-cadherin and higher expression of E-cadherin in presence of DDMSNP compared to the EMT induced or uninduced CSCs that were not treated with DDMSNP (Fig. 6Bi, ii). Furthermore, DDMSNP also reduced the expression of other EMT markers such as Snail, Slug, and Vimentin even when EMT was induced (Fig. 6Bi, ii). After transiently transfecting MDAMB-231 and 4T1 derived CSCs with E-cadherin, the effect of DDMSNP was rather noticeable, as it suppressed N-cadherin expression while increasing E-cadherin expression in comparison to non-transfected and transfected controls (Fig. 6Ci, ii). On the other hand, when specific siRNA caused decreased in E-cadherin expression compared to control, DDMSNP regained the expression when treated to both MDAMB-231 and 4T1 derived CSCs (Fig. 6Di, ii). In contrast, DDMSNP treatment reduced N-cadherin expression even when E-cadherin siRNA was present (Fig. 6Di, ii). Hence, all these data indicate that DDMSNP was able to control EMT induction by regulating the expressions of prominent EMT markers.

### Anti-metastatic effect of DDMSNP-loaded exosomes (e-DDMSNP) on CSC induced solid tumor-bearing BALB/c mice

**Fig. 7:**
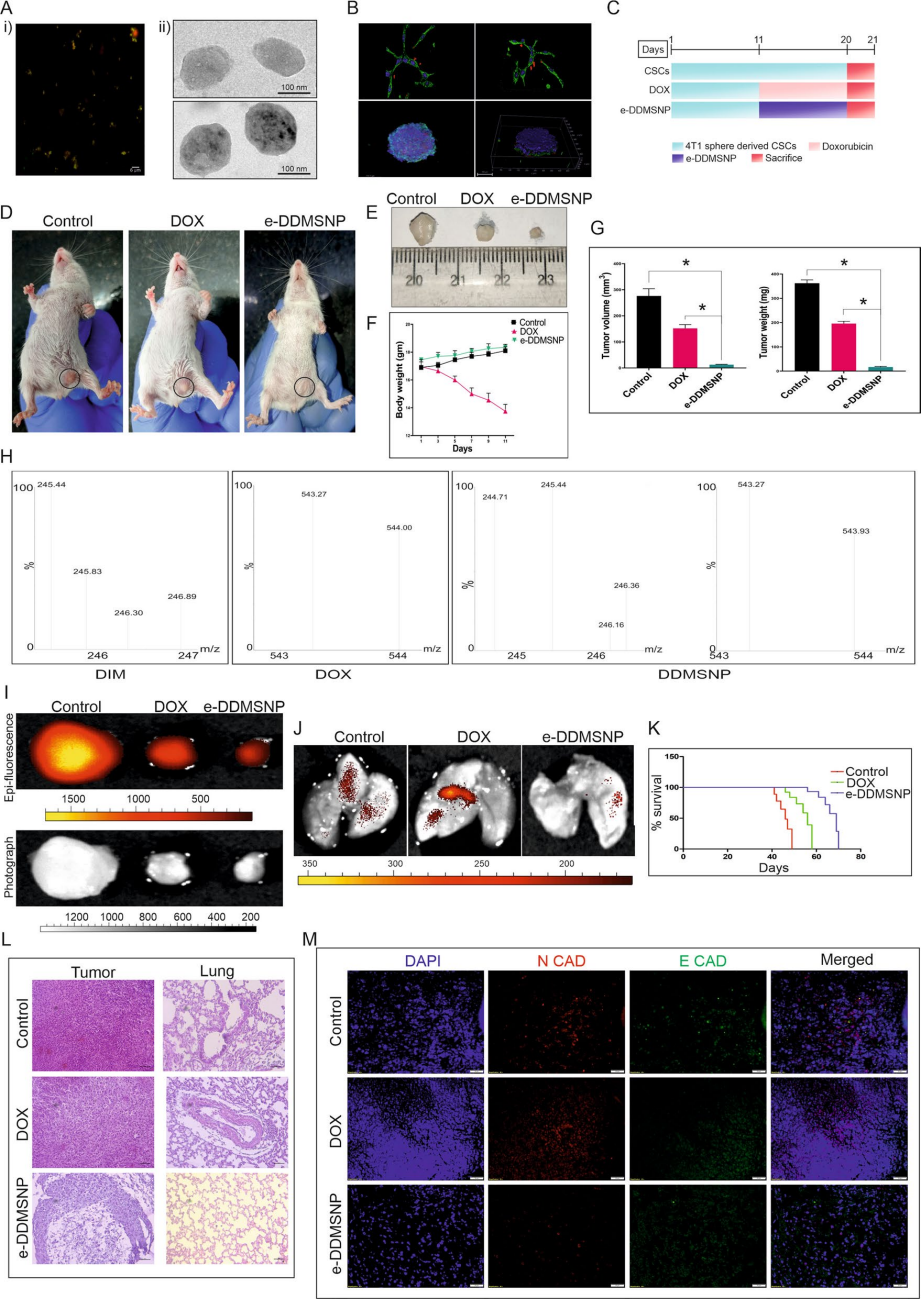
Anti-metastatic effect of e-DDMSNP on TNBC-CSCs derived tumors. **A** (i) Encapsulation of DDMSNP within exosomes, (ii) The morphology of an empty exosome and DDMSNP loaded exosomes captured by TEM. **B** Images from confocal microscopy represents the uptake of e-DDMSNP in cells (upper left panel) and sphere (lower left panel). Z-stacking analysis shows internalization of e-DDMSNP in cells (upper right panel) and in the sphere (lower right panel). **C** Schematic representation of experimental design to study the efficacy of DOX and e-DDMSNP in 4T1 CSC derived tumor-bearing mice. **D** Figure represents growth inhibition of tumors derived from 4T1 CSCs in BALB/c mice after DOX and e-DDMSNP treatment. **E** Figure shows decreased size of tumors in treated groups compared to untreated group. **F** Graph represents body weight changes during treatment. Data are expressed as the mean ± SD (n = 6). **G** Bar diagram represents tumor volume and tumor weight of control mice and mice receiving either DOX or e-DDMSNP (n = 6, mean ± SD), * denotes P < 0.0001. **H** Mass spectrometric analysis shows internalization of e-DDMSNP in tumors. Highest intensity peak in graphical representation denotes molecular mass of native DIM and native DOX. Graph shows same peak with highest intensity defining exact molecular mass of DIM and DOX in tissue extracts of e-DDMSNP treated tumors. **I** Fluorescence and bright field imaging represents tumor tissues of control, DOX and e-DDMSNP treated groups. **J** Fluorescence imaging of lung of treated and untreated groups indicates lung metastasis. **K** Effect of DOX and e-DDMSNP treatment on survivability of RFP expressing 4T1 CSCs derived solid tumor-bearing mice. Data are analyzed using Kaplan–Meier method. **L** Images denote H&E staining (× 20 ×  magnification) of tumors and lungs of treated and untreated groups. **M** Immunofluorescence assay represents the expressions of N-cadherin (red) and E-cadherin (green) in untreated control and DOX or e-DDMSNP treated 4T1 CSC derived solid tumors

In order to ensure biocompatibility, appropriate cellular uptake, preferred tumor homing and enhanced targeting efficacy, these DDMSNPs were encapsulated in exosomes that were extracted from cancer cells using an established procedure. The entrapment of DDMSNPs within the exosomes were further proved by fluorescent microscopy where co-localization of exosome marker CD63 labelled with green fluorophore (FITC) and red coloured DOX was noticed (Fig. 7Ai). Furthermore, TEM data suggested that the size of the e-DDMSNPs were 100–180 nm in diameter (Fig. 7Aii). Additionally, cellular uptake of this e-DDMSNPs was confirmed by confocal microscopy (Fig. 7B, upper panel), 3D imaging of which proved the proper binding and internalization of e-DDMSNPs in the TNBC cells (video 1). This proved the proper homing of these loaded exosomes in cells. Cellular uptake of e-DDMSNPs was further compared with native DIM, native DOX, nanotized DIM, nanotized DOX, DDMSNP1 and DDMSNP2 where most efficient compound uptake was noticed in case of e-DDMSNPs treated to MDAMB-231 and 4T1 cells (Fig. S5A). Confocal microscopy, on the other hand, also verified that e-DDMSNPs could penetrate the spheres' 3D cell mass, indicating that they could reach the core of the spheres, which resembled the tumor mass (Fig. 7B, lower panel; video 2).

Along with this, 4T1 sphere derived CSCs having stable RFP expression were injected to syngeneic BALB/c mice to form orthotopic breast tumors. These tumor models were developed to determine and compare the effect of e-DDMSNPs with DOX. Therefore, to comply this, the mice were treated with DOX and e-DDMSNPs for 10 days before being sacrificed. This treatment schedule was presented by schematic diagram in Fig. 7C. DOX, a well-known chemotherapeutic, was able to reduce tumor size after 10 days of therapy; however, the greatest reduction in tumor size was observed in the e-DDMSNP treated tumor group when compared to the control or DOX treated groups (Fig. 7D, E). Although, no significant differences in tumor size were observed in the empty nanoparticles (MSNPs) and empty exosomes treated groups as compared to the control groups (Fig. S5B-C). On the other hand, DOX administration significantly (P < 0.05) reduced the body weight of the host whereas minimal gain in body weight was observed in mice in both the control and e-DDMSNP-treated groups (Fig. 7F). In addition, a significant reduction in tumor volume and weight was noted in the e-DDMSNP treated group when compared to the control and DOX treated groups (Fig. 7G). All these data suggest prominent in vivo effect of e-DDMSNP. This was validated further by mass spectrometry, which verified the presence of DIM and DOX within the tumor niche of the e-DDMSNP treated group (Fig. 7H). As a result of this in vivo investigation, the existence of DIM and DOX in e-DDMSNP was confirmed once more. The most significant reduction in fluorescence intensity was seen after e-DDMSNP treatment in tumors derived from CSCs stably expressing RFP when compared to control or DOX treated tumors (Fig. 7I). This confirms restricted cell growth and division from CSCs upon e-DDMSNP treatment. As a consequence, least occurrence of lung metastasis was observed in e-DDMSNP treated RFP expressing tumor-bearing mice in contrast to control and DOX treated mice (Fig. 7J). Although, DOX was able to reduce tumor size, distance metastasis to lung and EMT induced phenomenon was increased in this group which was supported by the data of organ imaging (Fig. 7J). This again confirms the drawbacks of DOX when treated individually to TNBC. Nevertheless, DOX was found to be more effective than the untreated controls when the survival rate of these mice groups was taken into account over an extended period of time (Fig. 7K). However, the mice treated with e-DDMSNP had the highest effective survival rate (Fig. 7K). This was further supported when the mean survival time (MST), ILS (%), T/C (%) and TIR (%) were significantly (P < 0.05) increased in e-DDMSNPs treated mice as compared to solid tumor control group (Table 1). The combination treatment was found more effective than the monotherapy in suppressing the tumor volume and increasing life span of the tumor bearing host.

**Table 1:** Effect of e-DDMSNP on tumor volume and survivability of tumor-bearing mice

Groups	Tumor volume (mm^3^)	Tumor cell count (× 10^6^)	MST (days)	ILS (%)	T/C (%)	TIR (%)
Tumor control	245.310 ± 0.28	27.26 ± 1.2	44	–	–	–
4T1 CSC + exosomes	237.128 ± 0.25	26.85 ± 1.30	–	–	–	–
4T1 CSC + MSNPs	240.039 ± 0.20	27.28 ± 1.4	–	–	–	–
4T1 CSC + DOX	136.125 ± 0.22^#^	11.32 ± 0.7^#^	58^#^	42^#^	142^#^	42.13^#^
4T1 CSC + e-DDMSNP	13.662 ± 0.24*	3.8 ± 0.6*	75*	74*	174*	87.12*

Data were represented as mean ± SD, n = 6

^#^P < 0.05 significantly different from solid tumor control group

^*^P < 0.05 significantly different from DOX treated group. (one-way ANOVA followed by Tukey’s post hoc test)

The tissue architectures of tumors and lungs of the mice of these groups were further assessed by H & E staining. The observations in this staining confirmed maximum loss of cellular architectures and tissue compactness as well as appearance of vacuolar structures with necrotic regions in e-DDMSNP treated tissues compared to the untreated controls and DOX treated groups (Fig. 7L, left panel). Furthermore, the lung histology of the tumor-bearing mice treated with e-DDMSNP, DOX, and untreated control animals demonstrated the highest suppression of metastatic expansion following e-DDMSNP treatment (Fig. 7L, right panel). In addition, the lungs of tumor-bearing mice treated with e-DDMSNP did not exhibit the metastatic foci with increased cellular density and aberrant alveolar structures seen in the tissue fields of the untreated control and DOX treated groups. Instead, the lungs largely resembled normal lung tissue (Fig. 7L, right panel). However, empty MSNPs and empty exosomes could not alter the tissue architectures of TNBC tumors of mice, which proved specific effects of DIM and DOX combination in vivo (Fig. S5D). Along with this, e-DDMSNP did not show any organ toxicity in the host mice (Fig. S5E). This clarified the effectiveness of this exosomal nanoformulation against TNBC. Furthermore, the results of tissue immunofluorescence assays on tumor sections from untreated control, DOX treated, and e-DDMSNP treated groups corroborated all of these in vivo observations. Consistent with the in vitro findings, higher N-cadherin expression and decreased E-cadherin expression in untreated and DOX-treated tumors supported the notion that the beginning of EMT in these cancers caused them to metastasize to the lung (Fig. 7M). e-DDMSNP, on the other hand, not only decreased N-cadherin expression while increasing E-cadherin expression, but it also inhibited EMT induction by TNBC CSCs (Fig. 7M). Therefore, all these in vitro and in vivo findings suggest that e-DDMSNP is an efficient exosomal nanoformulation that may stop the advancement of DOX induced EMT in TNBC.

## Discussion

Recapitulation of ontogeny in cancer cells causes inappropriate activation of EMT [24]. The ability of cancer cells to spread is connected to transitions between epithelial and mesenchymal states [25]. EMT occurs in a fraction of cancer cells in primary tumors, resulting in enhanced migratory and invasive characteristics. This is the preliminary stage of metastasis and secondary tumor formation [24]. Therefore, in the context of the metastatic cascade, EMT is considered a crucial biological mechanism [24]. On the other hand, the phenotypic heterogeneity of CSCs presents a significant obstacle to the development of cancer therapies [26]. CSCs can modify their niche by manipulating several cell signalling pathways in order to sustain homeostatic processes such as EMT [27, 28]. Hence, there is a substantial correlation between CSC enrichment and EMT in tumor cell invasion and metastasis. The acquisition of chemo resistance in tumor cells is linked to these CSCs and EMT. In order to achieve safe and effective long-term responses, targeted therapies for tumor suppression should therefore be able to reach the entire tumor, including CSC niches, at any stage of the tumor’s development and eradicate both cancer cells and CSCs with adequate selectivity. This type of therapeutic modification is highly needed for TNBC, as current treatments for TNBC patients are limited compared with the non-TNBC patients having expression of ER, PR and/or HER2. Because of their inherent resistance, TNBC's CSCs cannot be treated with traditional chemotherapeutic drugs like DOX. This drug resistance of TNBC is highly associated with increased metastasis rate in patients [29]. Nonetheless, after treatment, remaining tumors exhibit CSC enrichment with all the characteristics of EMT [30]. As a result, TNBC patients have the lowest metastasis-free and overall survival rates of any breast cancer subtype [31–33]. They are also more likely to relapse and die within 5 years after first diagnosis and treatment [34]. Therefore, a novel beneficial strategy to combat this CSC mediated EMT progression in TNBC is highly needed. According to the National Comprehensive Cancer Network (NCCN) guidelines 2020, one of the most recommended treatment options for metastatic TNBC patient is DOX [35]. However, DOX, the first line chemotherapeutic against TNBC, not only develops drug resistance but also induces metastasis [36]. According to our results, DOX could increase the number of CSCs and caused overexpression of EMT promoting factors like N-cadherin, Snail, Slug and Vimentin as well as downregulation of epithelial markers like E-cadherin in CSCs (Fig. 2C–E). Similar to the hypothesis regarding DOX induced metastasis, we observed increased lung metastatic rate in the mice bearing tumors generated from 4T1 derived CSCs or 4T1 cells (Fig. 2F). Tumors generated in mice from 4T1-CSCs, on the other hand, had a higher metastatic rate than cancer cells (4T1), indicating that CSCs are more virulent in TNBC (Fig. 2F). Integration of phytotherapy and chemotherapy in multidrug treatment is a helpful strategy focused on toxicity reduction and dose adjustment of individual partner of the drug combination [37, 38]. Therefore, to overcome the effect of DOX on CSC mediated EMT induction a positive synergistic effect of phytochemical and conventional chemotherapeutic drug was investigated to achieve the best outcomes. For this reason, indole compound DIM was selected to investigate and analyse to confirm its synergistic effect with a focus on anti-TNBC strategies. The results not only show positive synergism between DIM and DOX but also confirm better anti-proliferative effect against TNBC cells compared to the individual treatment (Fig. 3). Anti-EMT effect of this combination was confirmed further by checking the expressional changes of proteins like N-cadherin, E-cadherin, Snail, Slug, and Vimentin in TNBC cells (Fig. 4A, B). However, effect of this combination on CSCs was further envisaged as CSCs are highly resistant to conventional chemotherapy [39]. Hence, to increase the effectiveness of this combination against CSC and to deliver DIM and DOX concomitantly both of them were encapsulated in single mesoporous silica nanoparticle. This was evolved as a formulation with sizes of ~ 100 nm, which is a favourable size for overcoming the hurdles of tight junction and drug efflux mechanism present in CSCs (Fig. 1) [40–42]. Furthermore, the prolonged release of DIM and DOX as a result of this nanoformulation kept these compounds active in the therapeutic range for a longer period of time (Fig. 1F). As expected, this DDMSNP was able to target CSCs by regulating their EMT promoting activity (Fig. 5). Therefore, DOX induced lung metastasis in TNBC tumor-bearing mice was restricted by this novel nanoformulation delivering both DIM and DOX to tumor niche developed from either CSCs or cancer cells (Fig. 5G). However, more aggressive lung metastasis from CSC-derived tumors compared to the cancer cell-derived tumors was also constrained by DDMSNP. Nonetheless, DDMSNP was also able to reduce forced EMT induction and overexpression of EMT markers in vitro (Fig. 6B–D)*.* Therefore, DDMSNP emerged as a prominent synthetic nanoformulation which supports DIM and DOX combination and co-delivery. However, a more potent drug delivery platform is thought to be biomimetic nanoparticles, which combine the unique features of natural biomaterials like exosomes with the functional versatility of synthetic nanoparticles [43, 44]. Hence, to increase the potency of DDMSNPs they have encapsulated in exosomes (Fig. 7A). Because of their biocompatibility, low toxicity, minimal immunogenicity, and stability in circulation, these exosomes have been employed as efficient nanocarriers [45–47]. Furthermore, this exosomal nanocarriers display efficient cellular uptake and target-homing capability which in turn might affect CSCs or cancer cells [45–47]. It is proved from the results that these e-DDMSNPs have maximum cellular uptake capacity compared to the native and nanoencapsulated DIM or DOX (Fig. S5A), as well as, they showed cellular homing in monolayer culture and also to the core of the spheres enriched with CSCs (Fig. 7B). Like DDMSNPs, e-DDMSNPs were also able to target CSCs efficiently and reduce CSC mediated progression of tumor growth in mice model. Furthermore, e-DDMSNPs inhibited CSC-mediated EMT progression and distant metastasis in vivo (Fig. 7I, J, L, M). Additionally, histological analysis after administration of e-DDMSNPs in repeated doses, neither tissue damage nor other abnormalities were detected in several major body organs. Hence, this study suggests that the e-DDMSNPs have promising potential candidate for combination treatment of TNBC by targeting CSC mediated EMT.

## Conclusion

The CSCs show a range of properties, some of which are indicative of EMT characteristics. CSCs contribute into metastatic progression followed by aggressive tumor behaviour, which may be one of the targets for anti-cancer therapy. Therefore, e-DDMSNP, or exosome sheathed mesoporous silica nanoparticle was synthesized to treat this EMT inducing CSCs of TNBC. Collectively, all results showed that e-DDMSNPs had a synergistic anti-tumor effect on TNBC cells and CSCs in vitro, and a significant anti-tumor effect in vivo. Although, this novel exosomal nanoformulation has great possibility for targeting CSCs, as well as EMT, further studies are warranted to elucidate the benefit-risk profile of e-DDMSNP for entering in the phases of clinical trial. Taken together, this evidence may pave the way for an effective approach to combat TNBC, providing that this strategy involves combination of both DIM and DOX.

### Supplementary Information

Supplementary Material 1.

Supplementary Material 2.

Supplementary Material 3.

## Data Availability

All the data generated or analyzed during the study are included in the article and can be produced whenever required.

## Abbreviations

TNBC: Triple negative breast cancer
EMT: Epithelial to mesenchymal transition
CSC: Cancer stem cell
DOX: Doxorubicin
DIM: 3,3′-Diindolylmethane
MSNP: Mesoporous silica nanoparticle
DDMSNP: DIM and DOX loaded MSNP
e-DDMSNP: DDMSNP-loaded exosome
CTAB: Cetyl trimethyl ammonium bromide
TEOS: Tetraethyl orthosilicate
APTES: 3-Aminopropyl triethoxysilane
NDIM: DIM encapsulated in MSNP
NDOX: DOX encapsulated in MSNP
DOX-fMSNPs: DOX-loaded amino functionalized MSNPs
SEM: Scanning electron microscope
TEM: Transmission electron microscope
DLS: Dynamic light scattering
FTIR: Fourier transform infrared spectrometer
PBS: Phosphate buffer saline
EGF: Epidermal growth factor
TPSA: Topological polar surface area
CI: Combination index
Fa: Fraction affected
DRI: Dose reduction index
DDMSNP1: DIM encapsulated in DDMSNP
DDMSNP2: DOX encapsulated in DDMSNP
MTT: 3-(4,5-Dimethylthiazol-2-yl)-2,5-diphenyl-tetrazolium bromide
PI: Propidium iodide
MST: Median survival time
ILS: Increase in life span
TIR: Tumor-growth inhibition rate
CES: CSC enriched sphere
BBB: Blood brain barrier
