# Supplementary material for: Exosome-sheathed porous silica nanoparticle-mediated co-delivery of 3,3′-diindolylmethane and doxorubicin attenuates cancer stem cell-driven EMT in triple negative breast cancer
Source: J Nanobiotechnology. 2024 May 25;22:285. doi: 10.1186/s12951-024-02518-0 (PMC11127288; doi:10.1186/s12951-024-02518-0)

**SUPPLEMENTARY INFORMATIONS:**

**Additional file 1:**

**Fig S1:** Impact of DOX on MDAMB-231 and 4T1 cell lines. A. i) Western blot shows expressions of EMT markers at various dosages of DOX treatment to MDAMB-231 and 4T1 cells. ii) Bar diagrams represent significant change in the expression of N-cadherin and E-cadherin compared to the control, * indicates P<0.05 and # indicates P<0.005. B. (i, ii) FACS analysis shows the expression of N-cadherin and E-cadherin in control and DOX treated groups. iii) Graphical representation depicts significant changes in protein expression in MDAMB-231 and 4T1 cells, * indicates P<0.005. No significant changes are observed in case of E-cadherin expression in both cell lines. C. (i, ii) FACS analysis represents comparison of E-cadherin expression between MDAMB-231 derived CSCs and MDAMB-231 cells.

**Fig S2:** Database based analysis of DOX’s drawbacks. A. Graph illustrates Kaplan-Meier Plotter Overall Survival (OS) (n=392) analysis in triple negative breast cancer harboring high level of n-cadherin and low level of E-cadherin at a follow up threshold of 200 months. Log-rank p-value is indicated within the boxes. B. Graph depicts TNM box plot of N-cadherin and E-cadherin gene expression in normal individuals, tumor tissue and metastatic tumor tissues of TNBC patients. C. ROC plotter represents the expression of N-cadherin and E-cadherin in DOX treated responder and non-responder groups.

**Fig S3:** CompuSyn analysis of NDIM, NDOX and DDMSNP. A. Figure shows dose effect plot of NDIM, NDOX and DDMSNP. B. CI plots presents CI values of <1 indicating synergism between DDMSNP1 (DIM) and DDMSNP2 (DOX). C. Isobologram represents effective doses required for inhibition at 50% (Fa 0.5), 75% (Fa 0.75) and 90% (Fa 0.9) for each individual drug. Synergism is demonstrated by the dose pair plotted as a point (symbol) below their respective Fa isobole or line. D. DRI of the drug combination of DDMSNP1 (DIM) and DDMSNP2 (DOX) is presented and DRI value >1 indicates favorable drug combination. E. All data are the representation of three independent experimental repeats. Fa, fraction affected; CI, combination index; DRI, dose reduction index. F. Cell viability assay demonstrates individual effects of NDIM and NDOX in MDAMB-231 and 4T1 cell lines after 24 hrs of treatment.

**Fig S4:** Effect of DDMSNP on MDAMB-231 and 4T1 cell lines. (A, B) The expressions of N-cadherin and E-cadherin in the control and DDMSNP-treated groups are assessed using flow cytometry. C. Graphical representation illustrates significant change in N-cadherin expression of MDAMB-231 cells and E-cadherin expression of 4T1 cells, * indicates P<0.005.

**Fig S5:** *In vitro* and *in vivo* proves of DIM and DOX nanoformulations. A. Bar diagrams represent cellular uptake of DIM, DOX, NDIM, NDOX, DDMSNP1 (DIM), DDMSNP2 (DOX), e-DDMSNP1 (DIM) and e-DDMSNP2 (DOX) at different time points. B. Graphical representation shows the experimental design to study the efficacy of empty MSNP and empty exosome. C. Figure represents the sizes of tumors of empty MSNP and empty exosome treated groups compared to the control. D. Images denote H&E staining (× 20X magnification) of tumors of untreated, empty MSNP and empty exosome treated groups. E. Images represent histology of body parts (heart, kidney, liver, spleen and lung) of untreated healthy control and e-DDMSNP treated tumor-bearing BALB/c mice.

**Video 1:** The representation of video captured by confocal microscopy indicates Z-stacking analysis of MDAMB-231 cells to ensure proper localization and internalization of e-DDMSNP.

**Video 2:** The video of Z-stacking analysis of MDAMB-231 derived CES depicts the proper binding and penetration of e-DDMSNP to the core of the sphere.

**Supplementary figures:**

**Fig. S1**


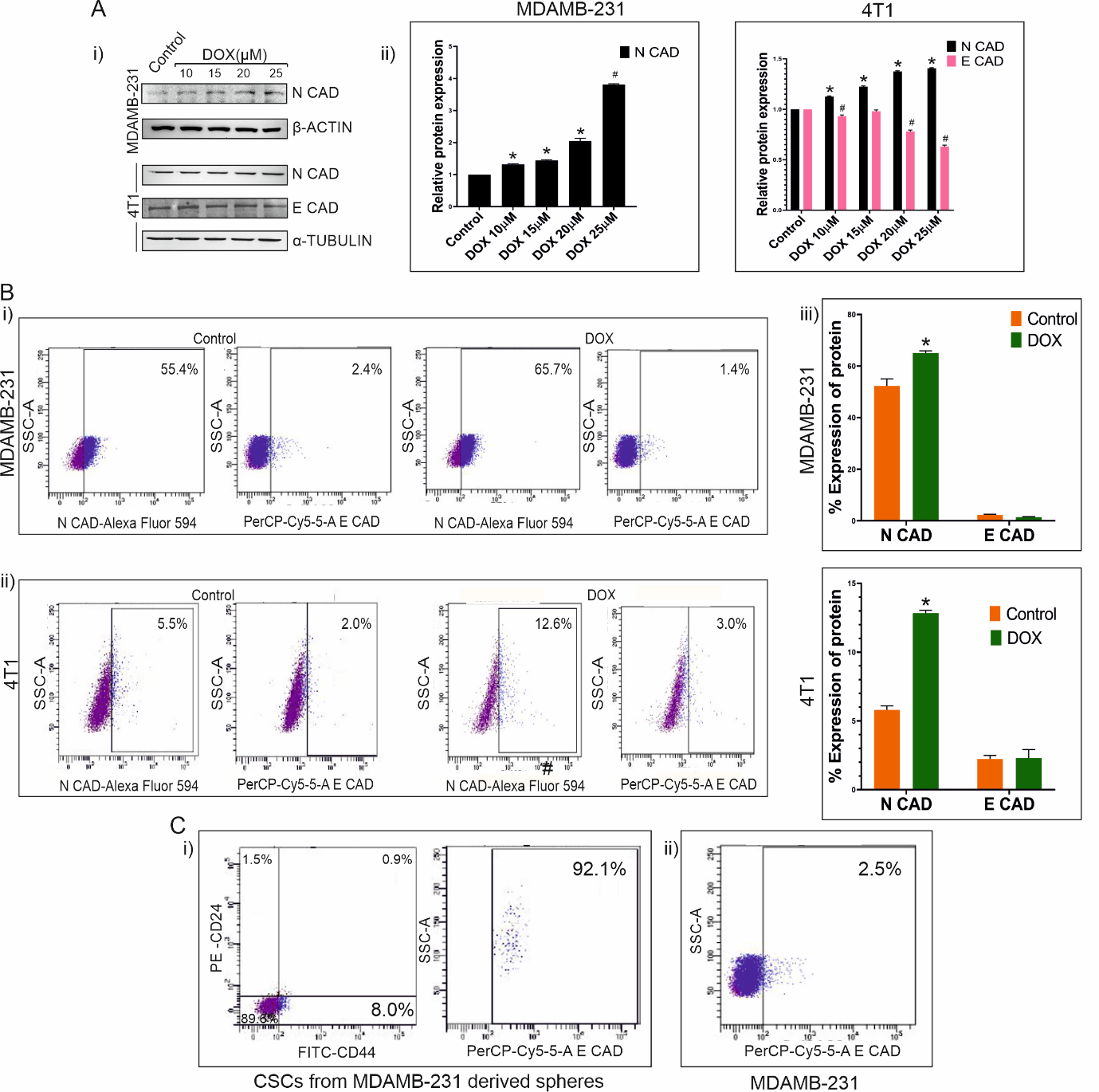


**Fig. S2**


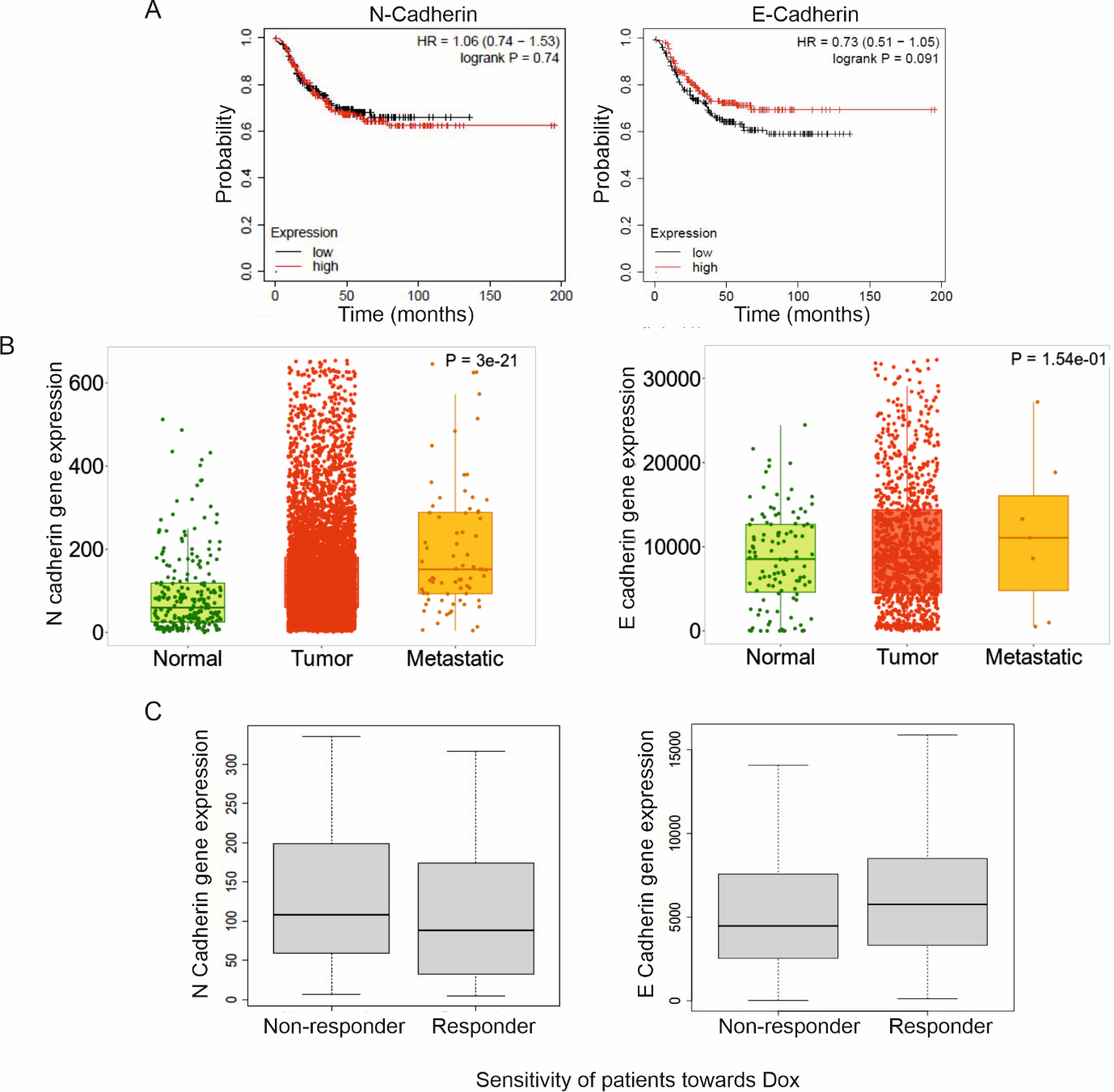


**Fig. S3**


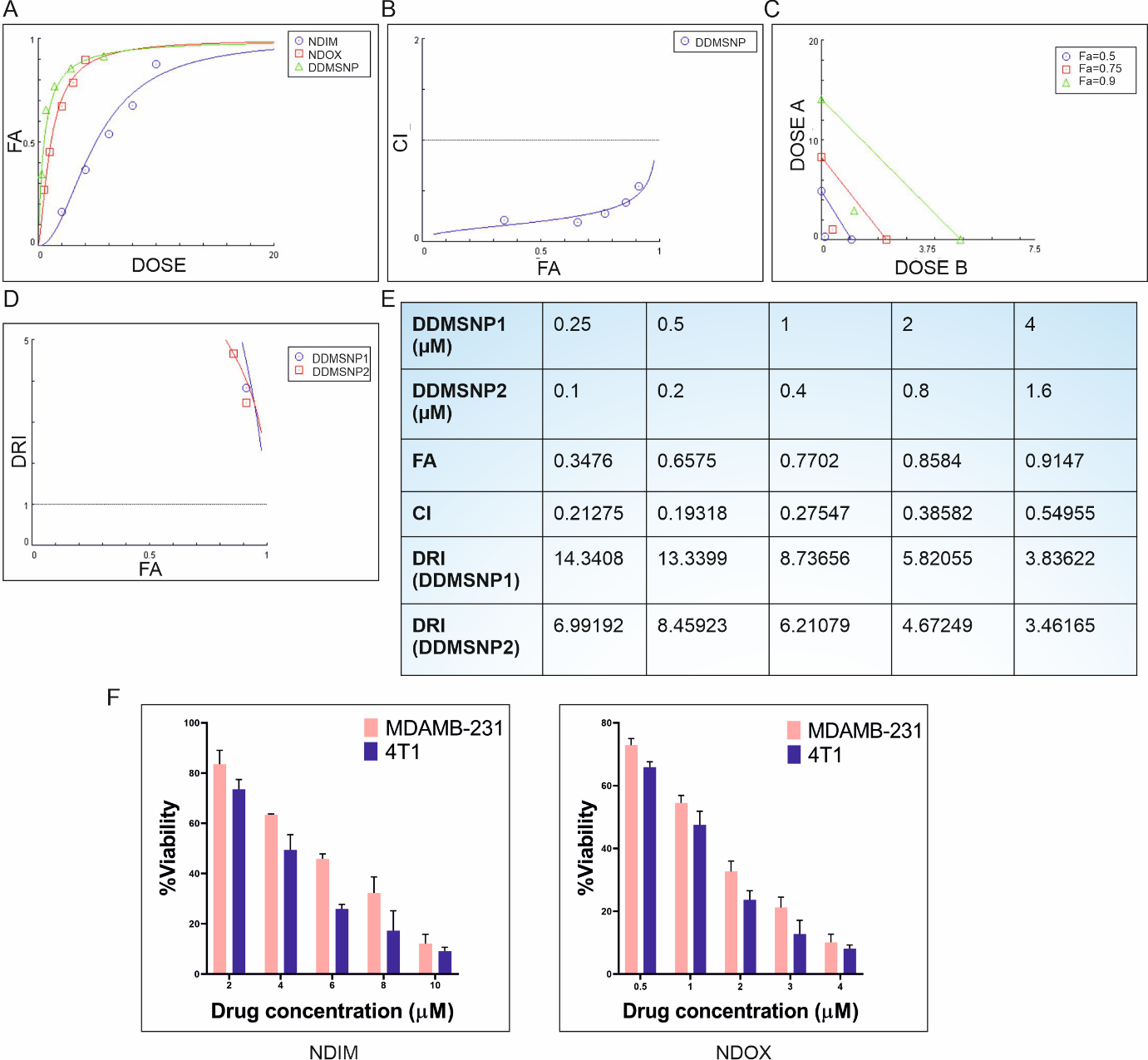


**Fig. S4**


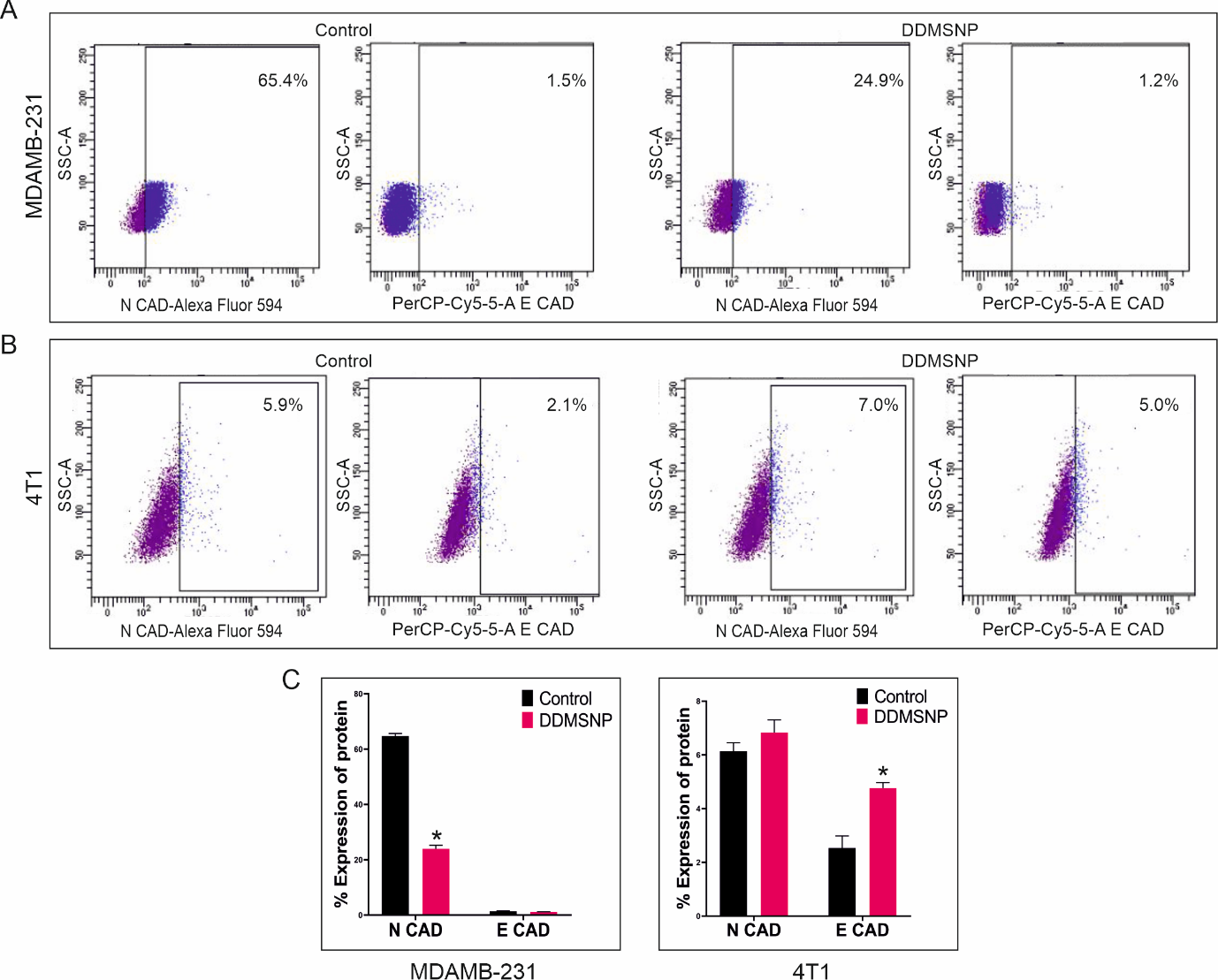


**Fig. S5**


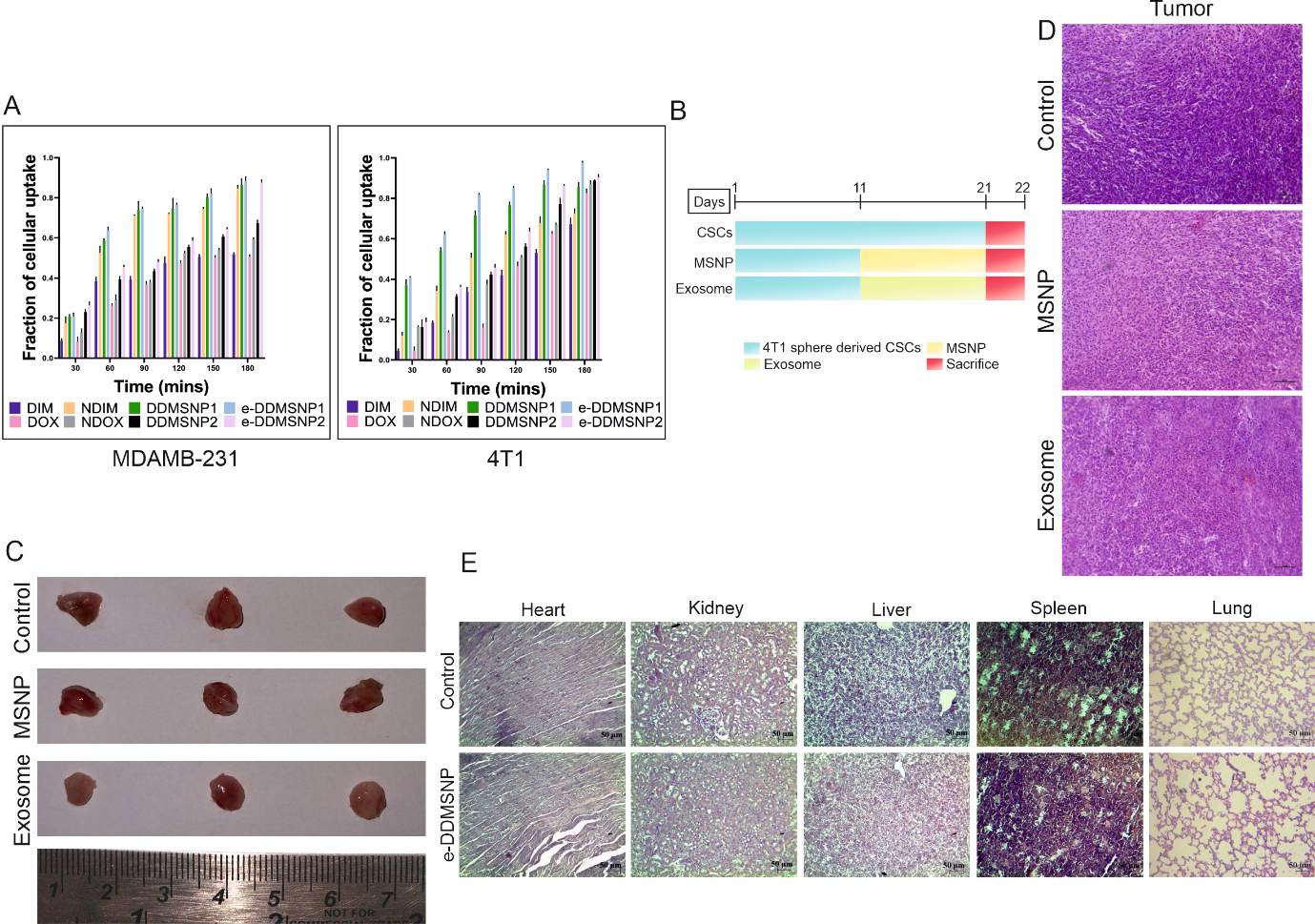

Supplement: Supplementary file 1 — Supplementary Material 1. [file 12951_2024_2518_MOESM1_ESM.docx]
